# A Quantitative Comparison of the Similarity between Genes and Geography in Worldwide Human Populations

**DOI:** 10.1371/journal.pgen.1002886

**Published:** 2012-08-23

**Authors:** Chaolong Wang, Sebastian Zöllner, Noah A. Rosenberg

**Affiliations:** 1 Department of Computational Medicine and Bioinformatics, University of Michigan, Ann Arbor, Michigan, United States of America; 2 Department of Biostatistics, University of Michigan, Ann Arbor, Michigan, United States of America; 3 Department of Biology, Stanford University, Stanford, California, United States of America; Dartmouth College, United States of America

## Abstract

Multivariate statistical techniques such as principal components analysis (PCA) and multidimensional scaling (MDS) have been widely used to summarize the structure of human genetic variation, often in easily visualized two-dimensional maps. Many recent studies have reported similarity between geographic maps of population locations and MDS or PCA maps of genetic variation inferred from single-nucleotide polymorphisms (SNPs). However, this similarity has been evident primarily in a qualitative sense; and, because different multivariate techniques and marker sets have been used in different studies, it has not been possible to formally compare genetic variation datasets in terms of their levels of similarity with geography. In this study, using genome-wide SNP data from 128 populations worldwide, we perform a systematic analysis to quantitatively evaluate the similarity of genes and geography in different geographic regions. For each of a series of regions, we apply a Procrustes analysis approach to find an optimal transformation that maximizes the similarity between PCA maps of genetic variation and geographic maps of population locations. We consider examples in Europe, Sub-Saharan Africa, Asia, East Asia, and Central/South Asia, as well as in a worldwide sample, finding that significant similarity between genes and geography exists in general at different geographic levels. The similarity is highest in our examples for Asia and, once highly distinctive populations have been removed, Sub-Saharan Africa. Our results provide a quantitative assessment of the geographic structure of human genetic variation worldwide, supporting the view that geography plays a strong role in giving rise to human population structure.

## Author Summary

The spatial pattern of human genetic variation provides a basis for investigating the history of human migrations. Statistical techniques such as principal components analysis (PCA) and multidimensional scaling (MDS) have been used to summarize spatial patterns of genetic variation, typically by placing individuals on a two-dimensional map in such a way that pairwise Euclidean distances between individuals on the map approximately reflect corresponding genetic relationships. Although similarity between these statistical maps of genetic variation and the geographic maps of sampling locations is often observed, it has not been assessed systematically across different parts of the world. In this study, we combine genome-wide SNP data from more than 100 populations worldwide to perform a formal comparison between genes and geography in different regions. By examining a worldwide sample and samples from Europe, Sub-Saharan Africa, Asia, East Asia, and Central/South Asia, we find that significant similarity between genes and geography exists in general in different geographic regions and at different geographic levels. Surprisingly, the highest similarity is found in Asia, even though the geographic barrier of the Himalaya Mountains has created a discontinuity on the PCA map of genetic variation.

## Introduction

The geographic structure of human genetic variation has long been of interest for its implications for studying human evolutionary history [1], [2], [3], [4], [5]. In recent years, the expansion of population-genetic datasets has contributed to an increase in geographic investigations of human genetic variation, often on the basis of classic multivariate statistical techniques such as PCA and MDS [6], [7], [8], [9], [10]. In PCA, samples are projected onto a series of orthogonal axes (principal components or PCs) that are constructed from a linear combination of genotypic values across genetic markers, such that each PC sequentially maximizes the variance among samples projected on it [11], [12]. Classic MDS analyzes a genetic distance matrix between pairs of samples and places the samples in a low-dimensional space in such a way that pairwise Euclidean distances among samples in the low-dimensional space approximate their relative genetic distances [13]. The population structure of genetic variation is often summarized in easily visualized two-dimensional statistical maps obtained from the first two components of PCA or MDS. Especially for large-scale single-nucleotide polymorphism (SNP) data, PCA and MDS are popular because of their computational efficiency and high level of resolution in decomposing the complex structure of human genetic variation [12], [14]. Generally, results produced by PCA and MDS are very similar to each other [15].

Several recent studies have reported detectable similarity between statistical maps of genetic variation and geographic maps of population locations. Such observations are particularly prominent within Europe, where striking similarity between genes and geography is observed both at a continental level [9], [16], [17] and in more localized studies such as in Finland [18], [19], Iceland [20], and Sweden [21]. Analogous but visually less striking observations have also been reported in studies of other geographic regions, including in worldwide samples [6], [7], [8], [10], [22], [23] and in samples from Asia [23], [24], [25], Africa [26], [27], China [28], [29], and Japan [30]. However, this similarity of genes and geography is in many cases reported in a qualitative sense and has not been assessed systematically across different studies, so that it has been difficult to compare levels of agreement between genes and geography in different regions. Further, different studies have used different sets of genetic markers and different statistical techniques (e.g. PCA and MDS), further complicating comparisons across datasets. Even for studies that used PCA, several versions of this technique have been employed in different studies. For example, some studies have performed PCA on genotypic matrices [9], [10], [12], [20], whereas others have applied PCA on pairwise genetic distance matrices [7], [22], [23].

A formal comparison of genes and geography in different regions using a single technique and a common marker set can provide a systematic basis for evaluating the role of geography in explaining the genetic similarity of individuals or populations in different locations. We have previously developed a Procrustes analysis approach to quantify the similarity between statistical maps of genetic variation and geographic maps [15]. This approach identifies data transformations that minimize the sum of squared Euclidean distances between two sets of coordinates while preserving relative pairwise distances among points within each set. The statistical significance of the similarity between genetic coordinates and geographic coordinates is then examined using a permutation test.

In this study, we apply the Procrustes approach together with PCA to systematically study the geographic structure of human genetic variation across different geographic regions. By compiling data from a variety of published sources [9], [23], [26], [31], [32], we have assembled genome-wide SNP data and geographic coordinates for 149 populations worldwide. Based on a common set of autosomal SNP markers shared by datasets collected from different studies, we evaluate the similarity between genes and geography in examples from Europe, Sub-Saharan Africa, Asia, East Asia, and Central/South Asia, as well as in a worldwide sample. We compare the level of similarity across the various datasets, finding that all show a high level of similarity, and that the highest similarity score appears in Asia. We also examine the dependence of the similarity on the choice of populations included in the analysis and on the number of markers studied. Our results provide information about the importance of geography in human evolutionary history, and can facilitate statistical methods for inferring the ancestral origin of human individuals from their genotypes.

## Results

We integrated published genome-wide SNP data on 4,257 individuals from 149 worldwide populations, taking data from the Human Genome Diversity Project (HGDP) [7], 31, International Haplotype Map Project Phase III (HapMap Phase III) [31], [33], and POPRES [9] samples, as well as from several other publications [23], [26], [32]. In our analyses, we focused on the data from 128 populations (Tables S1, S2, S3). We constructed six datasets for evaluating the geographic structure of genetic variation in different geographic regions: a worldwide sample, continental samples from Europe, Sub-Saharan Africa, and Asia, and subcontinental samples from East Asia and Central/South Asia (Table 1).

**Table 1:** SNP datasets for different geographic regions.

Region	Number of populations	Number of individuals collected	Number of high-missing-data individuals	Number of PCA-outlier individuals	Number of individuals in our analysis	Genotyping platforms	Data sources
Worldwide	53	938	0	0	938	Illumina 650 K	[31]
Europe	37	1,385	5	2	1,378	Affymetrix 500 K	[9]
Sub-Saharan Africa	23	356	6	2	348	Illumina 650 K; Illumina Human 1 M; Affymetrix NspI 250 K; Affymetrix 500 K; Affymetrix 6.0	[23], [26], [31]
Asia	44	760	0	11	749	Illumina 650 K; Affymetrix NspI 250 K; Affymetrix 6.0	[23], [31], [32]
East Asia	23	341	0	7	334	Illumina 650 K; Affymetrix NspI 250 K; Affymetrix 6.0	[23], [31], [32]
Central/South Asia	18	372	0	10	362	Illumina 650 K; Affymetrix NspI 250 K; Affymetrix 6.0	[23], [31]

Our analyses were based on 32,991 autosomal SNP markers that were shared among datasets obtained from different genotyping platforms. We applied PCA on datasets after quality control and removal of PCA outliers (see *Materials and Methods*), and we then used Procrustes analysis to compute the similarity score, denoted as 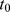, between the first two PCs of genetic variation and the geographic coordinates of the populations.

We evaluated the statistical significance of the similarity score by permutation. We further examined the robustness of our results using a leave-one-out approach, in which we repeated PCA and Procrustes analysis on datasets with a single population excluded. PCA coordinates obtained from these new datasets were compared to the original PCA coordinates obtained from the whole dataset and to the geographic coordinates, with the respective Procrustes similarity scores denoted as 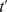 and 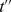 (see *Materials and Methods*). These analyses were applied systematically on all datasets.

### Worldwide sample

**Figure 1:**
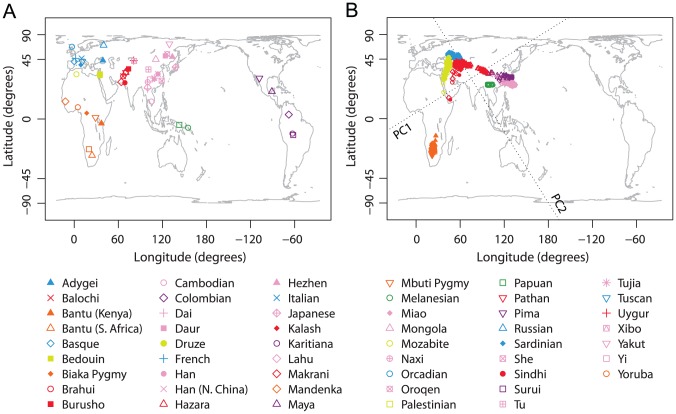
Procrustes analysis of genetic and geographic coordinates of worldwide populations. (A) Geographic coordinates of 53 populations. (B) Procrustes-transformed PCA plot of genetic variation. The Procrustes analysis is based on the Gall-Peters projected coordinates of geographic locations and PC1-PC2 coordinates of 938 individuals. The figures are plotted according to the Gall-Peters projection. PC1 and PC2 are indicated by dotted lines, crossing over the centroid of all individuals. PC1 and PC2 account for 6.22% and 4.72% of the total variance, respectively. The Procrustes similarity is 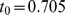 (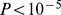). The rotation angle of the PCA map is 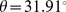.

Our worldwide example was based on 938 unrelated individuals from 53 worldwide populations (Figure 1A), taken from the study of Li *et al.* [7]. None of these individuals was found to have 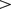
5% missing data or to appear as a PCA outlier.

A PCA plot finds that as in previous studies [7], [8], [10], samples from the same geographic region (indicated by colors in Figure 1) generally cluster together, and that different clusters align on the PCA plot in a way that qualitatively resembles the geographic map of sampling locations. The first two PCs of our PCA explain 6.22% and 4.72% of the total genetic variation, respectively. These values are considerably less than the values reported by Li *et al.* [7] in their Figure S3B, which were 52.3% for PC1 and 27.8% for PC2. The difference can be attributed primarily to the different versions of PCA used in the analyses. We applied PCA on the 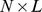 genotypic matrix for 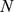 individuals and 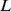 loci, whereas Li *et al.* applied PCA on an 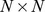 matrix recording levels of identity-by-state for pairs of individuals [7]. Although the two approaches provide visually similar PCA plots, the values and the interpretation of the proportions of variance explained by each PC differ, as they are based on quite distinct computations.

Using Procrustes analysis, we identified an optimal alignment of the genetic coordinates to the (Gall-Peters-projected) geographic coordinates that involved a rotation of the PCA plot by 31.91 counterclockwise. The genetic coordinates were then superimposed on the geographic map by applying the optimal transformation, thereby highlighting the similarity between genes and geography (Figure 1). This qualitative resemblance is demonstrated by the Procrustes similarity score of 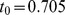, which is highly significant in 100,000 permutations (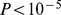). Applying the leave-one-out approach with populations excluded individually, the similarity score between genes and geography ranges from 0.697 to 0.715, with mean 0.705 and standard deviation 0.003 (Table S4). Some populations, such as Native American and Oceanian populations, align in Figure 1B distantly from their geographic locations. In most but not all cases, excluding one of these populations leads to an increase in the Procrustes similarity score.

### Europe

**Figure 2:**
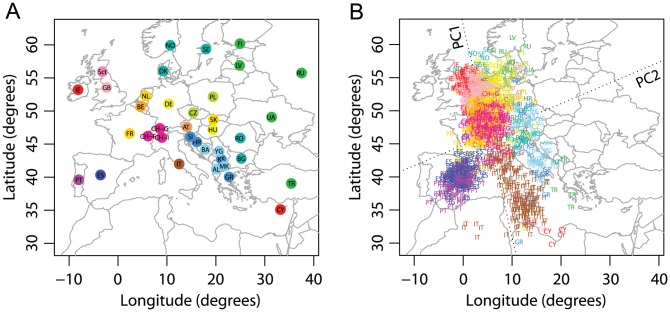
Procrustes analysis of genetic and geographic coordinates of European populations. (A) Geographic coordinates of 37 populations. (B) Procrustes-transformed PCA plot of genetic variation. The Procrustes analysis is based on the unprojected latitude-longitude coordinates and PC1-PC2 coordinates of 1378 individuals. PC1 and PC2 are indicated by dotted lines, crossing over the centroid of all individuals. Abbreviations are as follows: AL, Albania; AT, Austria; BA, Bosnia-Herzegovina; BE, Belgium; BG, Bulgaria; CH-F, Swiss-French; CH-G, Swiss-German; CH-I, Swiss-Italian; CY, Cyprus; CZ, Czech Republic; DE, Germany; DK, Denmark; ES, Spain; FI, Finland; FR, France; GB, United Kingdom; GR, Greece; HR, Croatia; HU, Hungary; IE, Ireland; IT, Italy; KS, Kosovo; LV, Latvia; MK, Macedonia; NL, Netherlands; NO, Norway; PL, Poland; PT, Portugal; RO, Romania; RU, Russia; Sct, Scotland; SE, Sweden; SI, Slovenia; TR, Turkey; UA, Ukraine; YG, Serbia and Montenegro. Population labels follow the color scheme of Novembre *et al.* [9]. PC1 and PC2 account for 0.30% and 0.16% of the total variance, respectively. The Procrustes similarity is 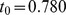 (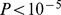). The rotation angle of the PCA map is 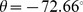.

Visually striking similarity betweeen PCA plots of genetic variation and a geographic map of Europe has been reported by several studies [9], [16], [17]. Our analysis was based on nearly the same sample studied by Novembre *et al.* [9], containing 1,385 individuals from 37 populations widely spread across Europe (Figure 2A). After excluding five individuals with 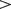
5% missing data and two PCA outliers, our final analysis examined 1,378 individuals.

Our PCA plot is very similar to the plot of Novembre *et al.* [9], with a close correspondence of genes and geography (Figure 2B). One difference is that in the PCA plot of Novembre *et al.* [9], individuals are more widely spread along PC2 than in our plot. As we applied PCA in the same way as Novembre *et al.* [9], the difference arises primarily because they employed coordinates given directly by the eigenvectors in PCA, such that PC1 and PC2 were scaled to have the same variance (J. Novembre, personal communication). To simplify the standardization of analyses across datasets, we chose not to scale the PC axes in our analyses, so that the relative amounts of genetic variation explained by each PC are reflected in the PCA plot (see *Materials and Methods*). Our PC1 and PC2 explain 0.30% and 0.16% of the total genetic variation respectively, in close agreement with the values of 0.30% and 0.15% reported by Novembre *et al.* [9].

We used Procrustes analysis to superimpose the PCA plot on the geographic map, rotating the PCA coordinates 72.66 clockwise (Figure 2). The rotated genetic coordinates of the European samples are spread over a larger distance along longitudinal lines than along latitudinal lines, although the geographic locations of the samples are distributed in the opposite way. This observation reflects the result that the genetic differentiation among Europeans is larger in a north-south direction than in an east-west direction [34]. The Procrustes similarity between the genetic coordinates and the geographic coordinates is 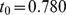 (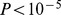). Excluding populations from the analysis individually, the Procrustes similarity between genes and geography ranges from 0.764 for the analysis without the United Kingdom to 0.810 for the analysis without Italy, with a mean of 0.780 across populations and a standard deviation of 0.007 (Table S5). Populations that have a relatively large effect on the similarity score are mostly those with large sample sizes (e.g., Italy, Portugal, Spain and United Kingdom). The Russian population is an exception; its sample size is small (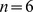), but the genetic coordinates of the Russian sample align poorly with the geographic coordinates [9] (Figure 2). Thus, this population has a relatively large effect on the similarity with geography (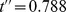 when excluding Russians, Table S5). Excluding Russians has minimal effect on the PCA coordinates for the remaining samples, however, as reflected in the high similarity score between the PCA coordinates before and after excluding the Russian sample (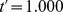, Table S5). Reducing the sizes of large samples also has a relatively small impact; when repeating our analyses on a subset of the data in which 50 individuals are selected randomly from populations with larger samples, 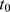 changes slightly to 0.777, and both 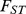 and the proportions of variance explained by PC1 and PC2 undergo slight increases (Figure S1).

### Sub-Saharan Africa

**Figure 3:**
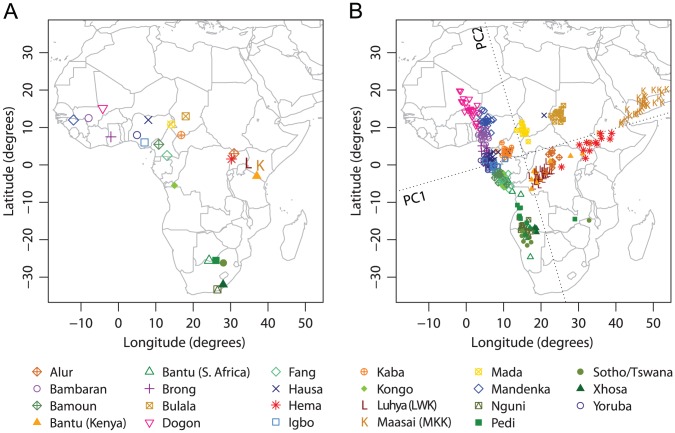
Procrustes analysis of genetic and geographic coordinates of Sub-Saharan African populations, excluding hunter-gatherer populations and Mbororo Fulani. (A) Geographic coordinates of 23 populations. (B) Procrustes-transformed PCA plot of genetic variation. The Procrustes analysis is based on the unprojected latitude-longitude coordinates and PC1-PC2 coordinates of 348 individuals. PC1 and PC2 are indicated by dotted lines, crossing over the centroid of all individuals. PC1 and PC2 account for 1.34% and 0.69% of the total variance, respectively. The Procrustes similarity is 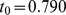 (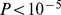). The rotation angle of the PCA map is 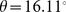.

Sub-Saharan Africa is the location of the origin of modern humans and has the highest genetic variation among all continents [7], [22], [35], [36], [37]. Previous studies have found that when isolated hunter-gatherer populations are included in the analysis, PCA plots of genetic variation in Sub-Saharan Africa display low qualitative similarity to the geographic map of sampling locations [7], [22], [38]. Bryc *et al.* recently studied 12 populations in West Africa, and revealed a high similarity between a SNP-based PCA map and the corresponding geographic map, when Mbororo Fulani, a nomadic pastoralist population, was excluded from the analysis [26]. By integrating SNP data from multiple sources [23], [26], [31], we investigated Sub-Saharan African populations in a broader region than in the analysis of Bryc *et al.* [26]. We first excluded four hunter-gatherer populations (!Kung, San, Biaka Pygmy, and Mbuti Pygmy) and Mbororo Fulani. After further excluding six individuals with 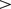
5% missing data and two PCA outliers, our analyses examined 348 individuals from 23 populations in Sub-Saharan Africa (Figure 3A).

Applying PCA on this combined Sub-Saharan African dataset, we found that PC1 accounts for 1.34% of the total genetic variation, largely separating populations from west to east. PC2 accounts for 0.69% of the total genetic variation and largely separates populations from north to south (Figure 3B). Generally, populations along the west coast of Africa cluster closely with each other, while interior populations form relatively isolated clusters. Bantu-speaking populations tend to cluster with each other, and can be divided into three groups according to their geographic locations: two populations in the west (Fang and Kongo), two in the east (Kenyan Bantus from the HGDP and Luhya), and five in the south (Southern African Bantus from the HGDP, Nguni, Pedi, Sotho/Tswana, and Xhosa). Despite the large geographic separation among these three groups, their genetic separation in the PCA plot is relatively small (Figure 3B). In particular, Luhya and Kenyan Bantus from the HGDP align between the western Bantu populations and the eastern non-Bantu populations such as Alur and Hema. The Maasai sample, consisting of 30 unrelated individuals randomly selected from the HapMap Phase III [31], [33], forms a cluster distant from the other populations along PC1 (and PC3, results not shown).

Procrustes analysis identifies a rotation angle of 16.11 counterclockwise for the genetic coordinates (Figure 3B), and the similarity score between genes and geography is 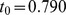 (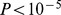). Among all populations, Maasai has the largest impact on both the PCA and Procrustes analysis (Table S6); as shown in Figure S2, when analyzed without Maasai, the other 22 populations align more closely with geography, and the Procrustes similarity score increases to 0.832 (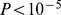). Excluding any of the populations in South Africa leads to a decrease of the similarity between genes and geography, and the lowest similarity is obtained when excluding the combined Sotho/Tswana sample (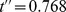, Table S6). This result suggests that the genetic map of Sub-Saharan Africans might look more similar to the geographic map if additional populations from the undersampled southern region of Africa were included.

When hunter-gatherer populations (!Kung, San, Biaka Pygmy, and Mbuti Pygmy) and Mbororo Fulani were included in the analysis, they appeared as isolated clusters on the PCA plots and greatly reduced the similarity between PCA maps and geographic maps (Figure S3, Table S7). The similarity score decreased from 0.790 to 0.548 after including all five of these populations in the analysis. This value, however, is still statistically significant, with a 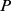-value of 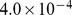; further, if we disregard the hunter-gatherer populations and Mbororo Fulani in Figure S3B and only examine the relative locations of the original 23 populations, we can still find a clear resemblance between genetic and geographic coordinates. Compared to the other 23 populations, the four hunter-gatherer populations appear as isolated groups at the south, and Mbororo Fulani appears at the north. These observations are clearer in plots with only one among the five outlier populations included at a time (Figure S3C–S3G), each of which also produces significant similarity scores between genetic and geographic coordinates (Figure S4, Table S7).

### Asia

**Figure 4:**
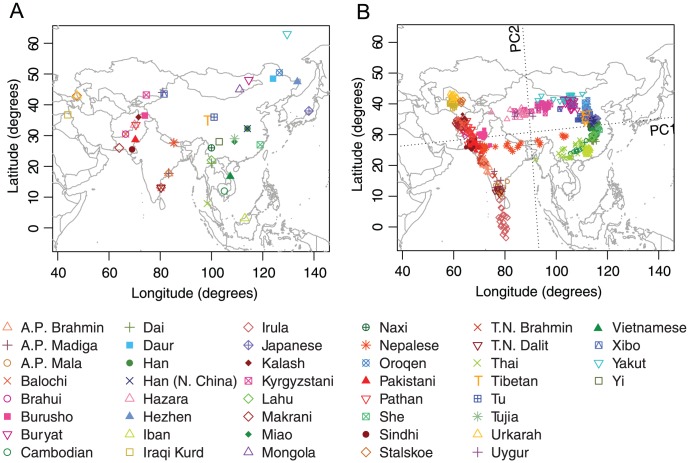
Procrustes analysis of genetic and geographic coordinates of Asian populations. (A) Geographic coordinates of 44 populations. (B) Procrustes-transformed PCA plot of genetic variation. The Procrustes analysis is based on the unprojected latitude-longitude coordinates and PC1-PC2 coordinates of 749 individuals. PC1 and PC2 are indicated by dotted lines, crossing over the centroid of all individuals. PC1 and PC2 account for 5.42% and 0.85% of the total variance, respectively. The Procrustes similarity is 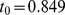 (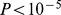). The rotation angle of the PCA map is 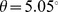.

Our Asian example included 760 individuals from 44 populations distributed widely across Asia (Figure 4A). Previous studies based on largely overlapping datasets have reported correlations between genetic and geographic distances across Eurasia [22], [23]. Our dataset combined data from these studies as well as from Li *et al.* [7] and Simonson *et al.* [32], and after excluding 11 PCA outliers, our final dataset for Asia contains 749 individuals.

In our PCA plot (Figure 4B), PC1 largely separates populations on different sides of the Himalayas, accounting for genetic variation in an east-west direction. PC2, on the other hand, distinguishes northern and southern populations. PC1 accounts for 5.42% of the total genetic variation, a much larger value than the 0.85% captured by PC2, reflecting large genetic distances between populations separated by the Himalayas. Interestingly, populations around the Himalayas form a ring shape on the PCA plot, with the Nepalese population from the Himalaya region aligning in the middle. As noted by Xing *et al.* [23], the Nepalese samples were collected from different subgroups that have different levels of ancestry shared with Central/South Asians and East Asians, and the dispersion of the Nepalese sample is therefore not unexpected. Tibetans, on the northern side of the Himalayas, do not spread over a large area in the plot and are well clustered with other East Asian populations.

One interesting result concerns the Uygur and Kyrgyzstani populations, both of which lie along ancient trade routes between Europe and East Asia. Compared to the Uygur population, which lies farther to the east, the Kyrgyzstani population clusters closer to East Asian populations, especially to the Yakut and Buryat populations, supporting a view that the Kyrgyzstani group has a proportion of its ancestry in Siberia [39]. A third population sampled from near the Uygur and Kyrgyzstani populations is the Xibo population, which clusters clearly with East Asians from northeastern China. This pattern matches the expectation given documentation that this Xibo group moved in 1764 from northeastern China to Xinjiang province [40], [41].

The PCA map of genetic variation in Asia is rotated 5.05 counterclockwise in the Procrustes superposition on the geographic map (Figure 4B). Despite the discontinuity caused by the Himalayas, most populations align in a way that is highly concordant with their geographic locations. This observation is confirmed by a Procrustes similarity score of 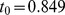 (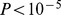). Among all populations, the tribal population Irula, which appears south of India as an isolated cluster in Figure 4B, has the largest impact among all populations on the Procrustes similarity with geography (Table S8). When excluding Irula, the PCA map aligns more closely with geography, with the Procrustes similarity increasing to 0.871 (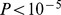, Figure S5). This exclusion generates increased separation on the PCA map for some populations. For example, in Figure S5, Iban from Sarawak is more clearly distinguished from other Southeast Asian populations. Overall, the similarity score between genes and geography in Asia is robust to the exclusion of any one population, with the lowest Procrustes similarity score of 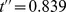 occurring when the Buryat population is excluded (Table S8).

### East Asia

**Figure 5:**
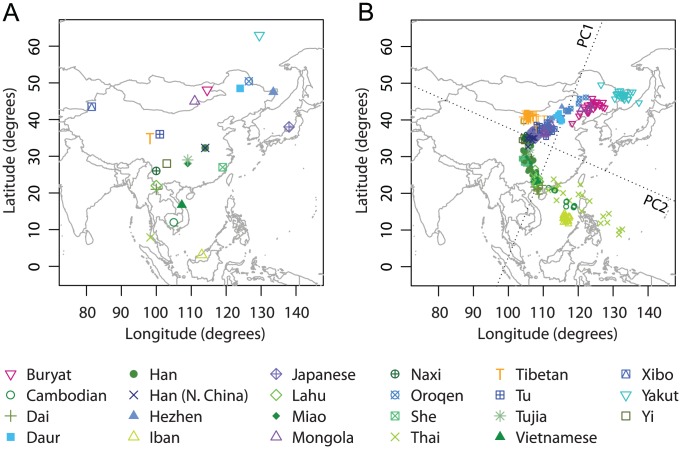
Procrustes analysis of genetic and geographic coordinates of East Asian populations. (A) Geographic coordinates of 23 populations. (B) Procrustes-transformed PCA plot of genetic variation. The Procrustes analysis is based on the unprojected latitude-longitude coordinates and PC1-PC2 coordinates of 334 individuals. PC1 and PC2 are indicated by dotted lines, crossing over the centroid of all individuals. PC1 and PC2 account for 1.58% and 0.98% of the total variance, respectively. The Procrustes similarity statistic is 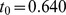 (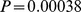). The rotation angle of the PCA map is 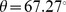.

To further examine populations on either side of the Himalaya Moutains, we performed additional analyses of East Asia and Central/South Asia. We first considered the East Asian populations in our Asian example. This dataset consists of 341 individuals from 23 populations. After excluding seven PCA outliers, our analyses were based on 334 individuals from 23 East Asian populations (Figure 5A).

Individuals in this East Asian dataset generally align along a curve on the PCA plot. PC1 explains 1.58% of the total genetic variation and largely accounts for a north-south genetic gradient; PC2 explains 0.98% of the genetic variation and mainly separates two Siberian populations (Buryat and Yakut) and three Southeast Asian populations (Cambodians, Iban, and Thai) from the other East Asian populations (Figure 5B). The Tibetan population is also separated by PC2, but on the opposite side to the Siberians and Southeast Asians. Overall, PC1 largely matches geography in the north-south direction, and PC2 shows only a partial similarity to the east-west direction.

The imperfect match between PCA coordinates and geography is reflected by a relatively low Procrustes similarity score of 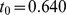, which, however, is still statistically significant with 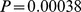. The optimal transformation rotates the PCA map 67.27 counterclockwise prior to superposition on the geographic map (Figure 5B). Interestingly, excluding populations one at a time, we found that the PCA coordinates were reflected over PC1 when Procrustes-transformed to match the geographic coordinates if either the Iban, Tibetan, or Yakut population was excluded (Figure S6). Such abrupt changes of the Procrustes transformation are consistent with the fact that PC2 matches less closely with geography; a reflection over PC1 has a small effect on the similarity score. The Procrustes similarity score with geography can be substantially increased by excluding Japanese (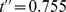, 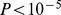); other than the Japanese population, Iban, Thai, and Yakut have the largest effect on the similarity scores both with geography and with the original PCA (Table S9).

### Central/South Asia

**Figure 6:**
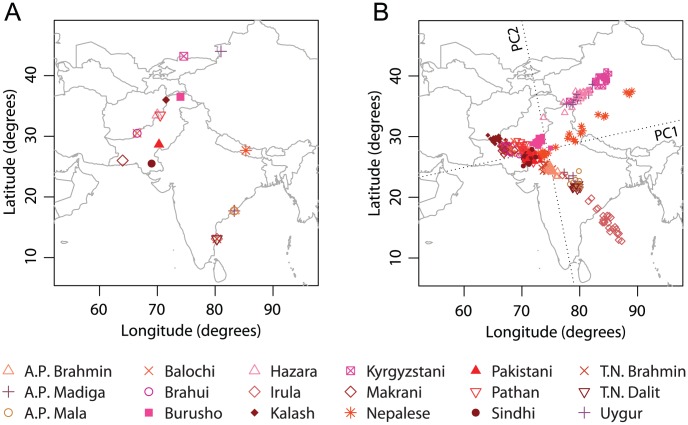
Procrustes analysis of genetic and geographic coordinates of Central/South Asian populations. (A) Geographic coordinates of 18 populations. (B) Procrustes-transformed PCA plot of genetic variation. The Procrustes analysis is based on the unprojected latitude-longitude coordinates and PC1-PC2 coordinates of 362 individuals. PC1 and PC2 are indicated by dotted lines, crossing over the centroid of all individuals. PC1 and PC2 account for 1.59% and 1.31% of the total variance, respectively. The Procrustes similarity statistic is 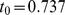 (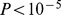). The rotation angle of the PCA map is 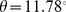.

Our last example focused on Central/South Asia, using an initial sample of 372 individuals from 18 populations. Ten individuals were excluded as PCA outliers, leaving 362 individuals from 18 populations for the final analysis (Figure 6A).

The first two components of the PCA anlaysis account for 1.59% and 1.31% of the total genetic variation, respectively. Overall, the PCA pattern for the separate anlaysis of Central/South Asian populations is similar to the pattern for the same set of populations in our analysis of all of Asia (Figure 4). After rotating the PCA coordinates 11.78 counterclockwise, we obtained a Procrustes similarity score of 0.737 (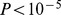) when comparing PCA coordinates to geography (Figure 6B). Most populations from Pakistan cluster closely on the first two PCs except for the Hazara population, which clusters with the Uygur population and aligns distantly from its sampling location. When excluding Hazara, the Procrustes similarity score to geography increases from 0.737 to 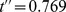, larger than for any other exclusion (Table S10). Excluding Irula has the second largest effect on the similarity score to geography, but more interestingly, this exclusion has the largest effect on the PCA coordinates (smallest value for 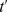 in Table S10). A closer examination of the PCA results reveals that when Irula is excluded, the Kalash population in Pakistan is separated from the other Pakistani populations and appears as an isolated group in the north (results not shown). This result accords with the identification of this isolated group as distinct in previous studies [8], [36].

### Comparison across geographic regions

**Figure 7:**
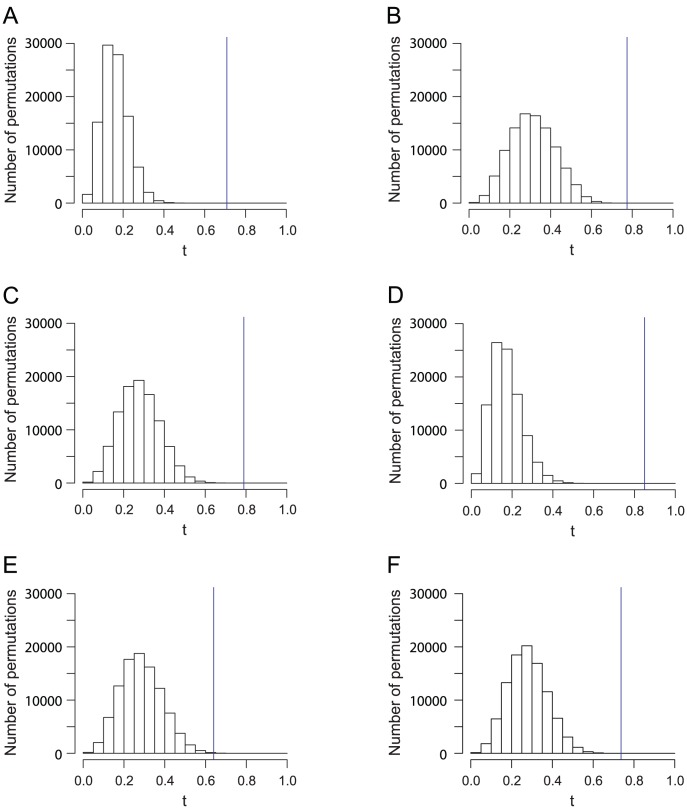
Histograms of the Procrustes similarity 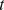 of 100,000 permutations for analyses in Figure 1, Figure 2, Figure 3, Figure 4, Figure 5, and Figure 6 **.** The blue vertical lines indicate the value of 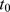. (A) The worldwide dataset in Figure 1 (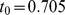, 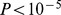). (B) The European dataset in Figure 2 (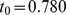, 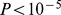). (C) The Sub-Saharan African dataset in Figure 3 (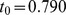, 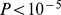). (D) The Asian dataset in Figure 4 (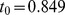, 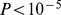). (E) The East Asian dataset in Figure 5 (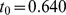, 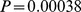). (F) The Central/South dataset in Figure 6 (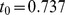, 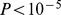).

We have found that significant similarity between genes and geography exists in general at different geographic levels (Table 2). The highest similarity score was found in the data from Asia, followed by Sub-Saharan Africa when five outlier populations were excluded, and by Europe. Five of the six analyses had 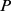-values smaller than 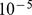, and only the data from East Asia had a nonzero 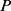-value in 100,000 permutations. When comparing the permutation distributions of the similarity score (Figure 7), however, a difference in the significance levels is evident for the five examples with 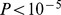. The worldwide and Asian datasets have similarity scores 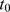 considerably exceeding the similarity scores from all 100,000 permutations (Figure 7A and 7D). By contrast, although the European, Sub-Saharan African, and Central/South Asian datasets have similarity scores higher than that of the worldwide dataset, their similarity scores are closer to the corresponding permutation distributions (Figure 7B, 7C, and 7F), indicating relatively high 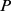-values compared to the worldwide data.

**Table 2:** Summary of the results for datasets from different geographic regions.

Region	Variance explained by PC1 (%)	Variance explained by PC2 (%)	Geographic map projection	Rotation angle 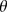 (  )	Procrustes similarity 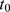	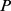 -value of 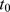	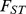 (%)
Worldwide	6.22	4.72	Gall-Peters	31.91	0.705	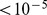	9.704
Europe	0.30	0.16	Unprojected	−72.66	0.780	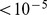	0.212
Sub-Saharan Africa	1.34	0.69	Unprojected	16.11	0.790	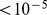	1.334
Asia	5.42	0.85	Unprojected	5.05	0.849	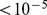	4.706
East Asia	1.58	0.98	Unprojected	67.27	0.640	0.00038	1.874
Central/South Asia	1.59	1.31	Unprojected	11.78	0.737	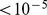	2.140

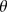
 is the rotation angle for the PCA map that optimizes the Procrustes similarity with the geographic map, and it is measured in degrees counterclockwise. 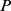-values are obtained from 100,000 permutations of population labels.

**Figure 8:**
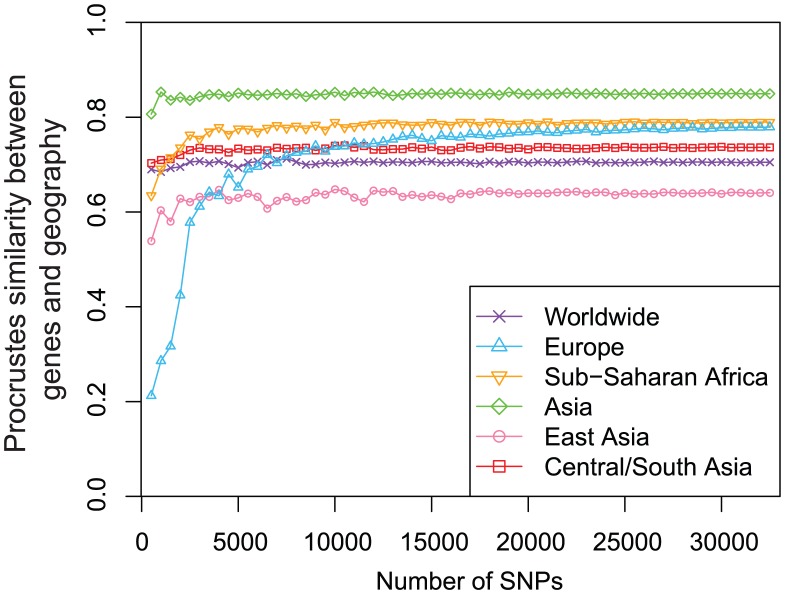
Procrustes analyses of genetic and geographic coordinates based on different numbers of loci. The same sets of 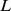 randomly selected markers were used to generate PCA maps of genetic variation to compare with geographic maps for different regions. 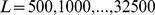.

To examine the robustness of our results to the number of SNPs analyzed, we repeated our analyses with subsets of randomly selected loci. We found that our Procrustes similarity scores between genes and geography are quite robust as long as enough SNPs (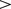
10,000) are used (Figure 8). Indeed, for the worldwide and Asian datasets, 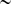
1,000 SNPs are sufficient to obtain a similarity score between genes and geography close to the score obtained using all 32,991 SNPs. For the African, East Asian, and Central/South Asian datasets, the number of SNPs needed increases to 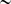
4,000. Interestingly, many more SNPs are required for the European dataset to reach a high similarity score between genes and geography. Although the increase of the similarity score for the European dataset becomes slow when the number of SNPs exceeds 10,000, it continues even when the number of SNPs is as high as 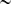
30,000 (Figure 8). If we use the same 197,146 SNPs as used by Novembre *et al.* [9], the similarity score between genes and geography for the European example would become 0.799, slightly higher than the value for our Sub-Saharan African example based on 32,991 SNPs. This larger number of SNPs required might reflect a relatively homogeneous population structure in Europe that requires more genetic markers to characterize subtle differentiation.

**Figure 9:**
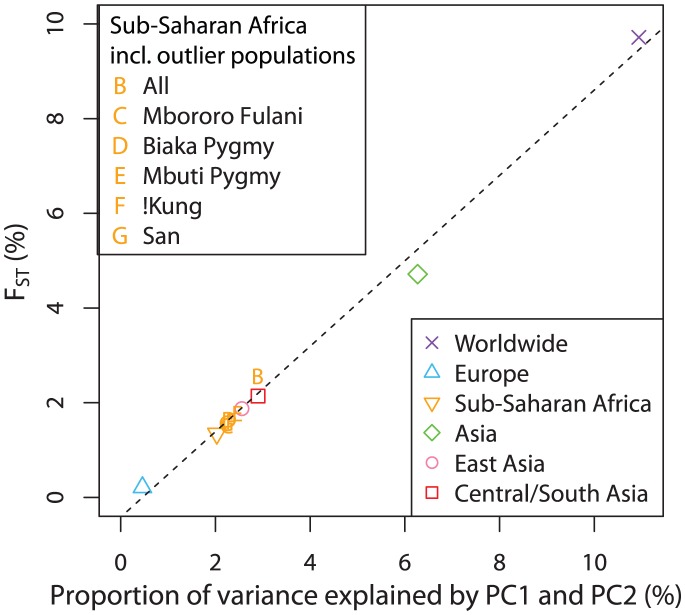
Relationship between 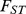 and the proportion of genetic variation explained by the first two components of the PCA. Both the main analyses of the paper in Table 2 and the supplementary analyses of Sub-Saharan Africa, in which certain populations excluded from the main analysis are included, are considered in obtaining the regression line. The values on the x-axis were obtained by summing the proportions of variance explained by PC1 and PC2 (columns 2 and 3 in Table 2, columns 6 and 7 in Table S7). 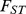 values were estimated from the same datasets as used in the PCA (column 7 in Table 2, column 11 in Table S7). The dashed line indicates the linear least squares fit of 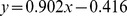. The Pearson correlation is 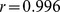.

To explore the relationship between genetic differentiation and the number of SNPs required to produce convergence in the Procrustes similarity, we computed 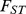 across populations, a measurement of population differentiation, for all of our datasets, on the basis of the 32,991 autosomal SNP markers. We found 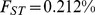 for the European dataset, much smaller than the values of 9.704% and 4.706% for the worldwide and Asian datasets. The values of 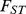 for the Sub-Saharan Africans (without outlier populations), the East Asians, and the Central/South Asians are 1.334%, 1.874% and 2.140%, respectively. As expected, datasets that have less population differentiation, as indicated by smaller 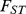 values, need more markers to reveal geographic structure in the PCA plot, consistent with a previous finding that the dataset size required for the population structure to be evident in PCA is inversely related to 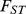 [12]. Further, we found 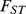 and the sum of the proportions of variance explained by PC1 and PC2 to be positively correlated (Pearson correlation 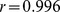, Figure 9). This strong linear correlation is not surprising because of the connection between 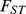 and the proportions of variance: 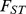 can be computed as the proportion of the variance in an allelic indicator variable contributed by between-population differences [42]. It has been shown under a two-population model that the proportion of the total variance explained by PC1 is approximately equal to 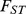 [43]. Here, we have observed a qualitatively similar relationship.

## Discussion

Both simulation-based and theoretical studies have shown that under spatial models in which migration and gene flow occur in a homogeneous manner over short distances, a similarity between PCA maps of genetic variation and geography is predicted [43], [44]. In this study, we have systematically assessed this similarity in different geographic regions using a shared set of autosomal SNPs and a shared statistical approach. We have found that although they generally explain a relatively small proportion of the total genetic variation, the first two principal components in PCA often produce a map that resembles the geographic distribution of sampling locations. Our results quantitatively demonstrate the general existence in different geographic regions of a considerable similarity between genes and geography, supporting the view that geography, in the form of incremental migration and gene flow primarily with nearby neighbors, plays a strong role in producing human population structure.

One particularly interesting observation concerns our analysis of the Asian dataset. Asia contains the Himalaya region, a strong geographic barrier to gene flow that has generated noticeable genetic differentiation between populations on opposite sides [45]. Such barrier effects can produce a distortion of PCA maps from those expected under homogeneous isolation-by-distance models [43], [44], leading to a decrease in the similarity to geography. However, although the concordance of a PCA plot with geography is perhaps best known for Europe — which does not have a barrier of comparable importance to the Himalayas — we obtained the unexpected result that in spite of the Himalaya barrier, the Procrustes similarity score 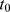 was actually highest in Asia. When further examining the population structure on separate sides of the Himalayas, we found lower similarity scores between genes and geography in our East Asian and Central/South Asian samples. Especially for the East Asian sample, our results indicate weaker correlation between genes and geography in the east-west direction.

To make the similarity scores between genes and geography commensurable for different datasets, we performed our analyses with the same markers and the same statistical approach. However, one aspect of the analysis that is not homogeneous across datasets is the nature of the geographic coordinates. For example, while most of the analyses employed population sampling locations, for the European dataset, coordinates did not necessarily represent sampling locations. Sampling locations may also vary in the extent to which they represent long-term locations where groups have resided. One example that highlights this issue is the Xibo population, which was sampled in northwestern China, but which clusters genetically with populations in northeastern China (Figure 5). This group is known to have migrated westward from near Shenyang in northeastern China about 250 years ago [40], [41], and if we were to use the coordinates of Shenyang (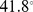
N, 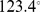
E) for Xibo rather than the sampling location, 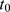 would increase from 0.640 to 0.654 for the East Asian dataset, from 0.849 to 0.859 for the Asian dataset, and from 0.705 to 0.709 for the worldwide dataset.

Additional limitations apply to our geographic analysis. In all of the datasets, population-level rather than individual-level coordinates were used, so that all individuals from the same population were assigned to a single geographic location. This approach can potentially obscure substructure within populations. For example, although both the northern and southern Han Chinese groups from the HGDP dataset were assigned to the same location, they can be genetically distinguished from each other, with the northern group clustering closer to the northern populations in China (Figure 5). Use of individual-level coordinates might lead to higher values of the similarity score 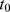. Another concern is that the choice of a map projection (including the projection that consists of using unprojected latitudes and longitudes as a rectangular coordinate system) can have different effects in geographic regions at different distances from the equator, as the level of distortion of the surface of the earth varies with the choice of projection. This issue is expected to be of greatest concern in analyses at high latitudes or in datasets with a wide range of latitudes.

We note that theoretical work and simulation studies have found that results from the PCA approach can be sensitive to the sample size distribution over geographic space [43], [44], [46]. In most of our analyses excluding one population at a time, patterns in PC1 and PC2 did not differ greatly from analyses in which all populations were included. However, exclusions of genetically distinctive populations, populations that were geographically distant from the center of a dataset, or populations with large sample sizes sometimes had sizeable effects on 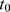. In some analyses, particularly in considering the Luhya and Maasai populations from the HapMap, we therefore included only a subset of available individuals in order to reduce the influence of the large sample sizes for these populations. More generally, an analysis of the role of the geographic distribution of the sample can be performed by analysis of subsamples of a full dataset with different levels of geographic unevenness. A previous analysis of population structure inference using *STRUCTURE* for a variety of samples with different geographic distributions did not find a particularly strong role for the geographic dispersion of the sample [47], but the issue has not yet been systematically investigated with PCA.

Through a combination of PCA and Procrustes analysis, we have investigated genes and geography using the same standardized approach in different regions. The general observation of a concordance of genes and geography in different regions and at different geographic levels can provide a foundation for refinement of methods for inferring local geographic origin of human individuals from their genotypes [e.g. 9], [19], [48]. In addition, our computations illustrate the use of Procrustes analysis in assisting the interpretation of PCA, such as in comparing PCA maps to different types of spatial maps and in assessing the impact of certain populations or individuals on PCA results. Similar applications of PCA and Procrustes approaches can be used to evaluate evolutionary models by comparing PCA maps obtained from observed data to those obtained from simulated data generated by these models. With the incorporation of the Procrustes similarity score for quantifying patterns in PCA, results from PCA can potentially find new uses in additional applications in population-genetic studies.

## Materials and Methods

### Genotype data

We examined genome-wide SNP datasets previously reported in several studies [9], [23], [26], [31], [32]. The data of Pemberton *et al.* [31] merged unrelated samples from earlier datasets obtained from the HGDP [7] and HapMap Phase III [33], [49]. Some of the data of Xing *et al.* [23] were previously reported in an earlier paper of Xing *et al.* [22].

Because the datasets were genotyped on different genotyping platforms, including Illumina 650 K [31], Illumina Human 1 M [31], Affymetrix 500 K [9], [26], Affymetrix NspI 250 K [23], and Affymetrix 6.0 [23], [31], [32], we identified a shared set of 32,991 autosomal SNPs included in all five datasets [9], [23], [26], [31], [32]. This number was smaller than the maximum possible set of overlapping SNPs shared among these genotyping platforms, because some SNPs were excluded during the quality control procedures of the studies that originally published the data [9], [23], [26], [31], [32]. At 6,549 among these 32,991 markers, the datasets from Novembre *et al.* [9] and Bryc *et al.* [26] had genotypes given for opposite strands when compared to the datasets of Xing *et al.* [23], Pemberton *et al.* [31], and Simonson *et al.* [32]. In these instances, we converted the genotypes from Novembre *et al.* [9] and Bryc *et al.* [26] to the opposite strand, so that genotypes were consistent across datasets from different sources. In total, we obtained genotype data on 32,991 autosomal SNPs for 4,257 samples from 149 populations worldwide, with dense sampling from Asia, Europe, and Sub-Saharan Africa. In our final dataset, the physical distance between pairs of nearby SNPs has mean 84 kb (median 45 kb).

We next created six datasets at different geographic scales, including a worldwide sample, continental samples for Europe, Sub-Saharan Africa, and Asia, and subcontinental samples from East Asia and Central/South Asia (Figure S7, Table 1). For the worldwide example, we included 938 unrelated individuals from 53 populations in the HGDP [7], [31]. For the European sample, we used a set of individuals that was nearly identical to that analyzed by Novembre *et al.* [9], containing 1,385 individuals from 37 populations defined by ancestral origins. We did not include two French individuals (sample ID 31645 and 32480) that were included by Novembre *et al.* [9] but that are not found in the release we obtained of the POPRES dataset in the NCBI dbGaP database [50], [51]. For Sub-Saharan Africa, we integrated data on African populations from three sources [23], [26], [31], including 30 unrelated Luhya (LWK) individuals and 30 unrelated Maasai (MKK) individuals, both randomly selected from the HapMap Phase III [31]. Because some populations in Sub-Saharan Africa are known to be genetically distinctive when compared to most other Sub-Saharan Africans [7], [8], [23], [26], [36], [37], we created two datasets for Sub-Saharan Africa, one including and the other excluding these distinctive populations (!Kung, San, Biaka Pygmy, Mbuti Pygmy, and Mbororo Fulani). When excluding all five of these populations, we have 356 individuals from 23 Sub-Saharan African populations. Including them, we have 422 individuals from 28 groups. Note that both Pygmy populations that we examined are from the HGDP [7], [31], and we did not include the Mbuti Pygmy data from Xing *et al.* [23]. Further, we also did not include the Luhya individuals from Xing *et al.* [23]; these individuals are a subset of those of the HapMap [31], [33]. As in Xing *et al.* [23], we analyzed three Sotho samples and five Tswana samples together as a single population, labeled as “Sotho/Tswana.”

Our sample from Asia has 760 individuals from 44 populations with sampling locations distributed widely across Asia. These data include 27 populations from the HGDP dataset [7], [31], 16 populations from Xing *et al.* [23], and one population (Tibetan) from Simonson *et al.* [32]. For populations studied by both Pemberton *et al.* [31] and Xing *et al.* [23] (Cambodian, Han Chinese, and Japanese), we only included the HGDP samples from Pemberton *et al.* [31]. Samples for East Asia and Central/South Asia are subsets of the Asian sample. The East Asian sample consists of 341 individuals from 23 populations: 18 populations from the HGDP dataset [7], [31], 4 populations from Xing *et al.* [23], and the Tibetan population from Simonson *et al.* [32]. The Central/South Asian sample has 372 individuals from 18 populations in total, including 9 populations each from the HGDP dataset [7], [31] and the Xing *et al.* dataset [23].

We applied two additional processing steps on each dataset to remove samples with high missing data rates and samples that appear to be outliers. First, we removed individuals with more than 5% missing data in the 32,991 SNPs. Next, in each analysis, we used an iterative PCA approach to identify and remove outlier individuals, as outliers can potentially distort PCA maps of genetic variation [52]. After applying PCA on a dataset, individuals greater than 10 standard deviations from the mean PC position on at least one of the top 10 PCs were considered outliers and were removed from the dataset. This procedure was repeated iteratively until no more outliers were detected. For all datasets, only a small proportion of samples were identified as outliers and removed by this procedure (Table 1). The data processing procedures are illustrated in Figures S7, S8, S9, and are summarized in Table 1. Individuals that were identified as PCA outliers are listed in Table S11.

### Geographic coordinates

We assigned all individuals from the same population to a single geographic location, as listed in Tables S1, S2, S3. For the HGDP samples [31], we used previously reported coordinates as the geographic locations for all populations (Table 1 in [45]). The geographic locations for the European dataset were reported in Table S3 of Novembre *et al.* [9], and represent countries of origin. The geographic coordinates for the African populations from Bryc *et al.* [26] are sampling locations, and we used the values reported by Tishkoff *et al.* [37] in their Table S1. Geographic coordinates for populations from Xing *et al.* [23] were kindly provided by J. Xing. For the Tibetan samples, we used the sampling location reported by Simonson *et al.* [32]. For the two HapMap populations included in this study (Luhya and Maasai), we used the sampling locations reported by HapMap [33].

We used longitude and latitude measured in degrees as our geographic coordinates 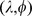 for all datasets except the worldwide dataset. Latitudes in the southern hemisphere and longitudes in the western hemisphere were denoted by negative values. For the worldwide dataset, we shifted the Americas by adding 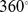 to longitudes smaller than 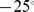. We then used the Gall-Peters projection, an equal-area projection that preserves distance along the 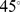
N parallel, to obtain rectangular coordinates 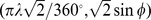 as our geographic coordinates. For other datasets, we used unprojected longitude-latitude coordinates.

### Principal components analysis

We coded the genotype data for each dataset by an 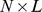 matrix 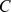, in which 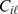 counts the number of copies of a reference allele at locus 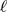 of individual 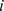, 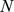 is the number of individuals, and 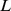 is the number of loci. For autosomal SNPs, 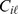 is 0, 1, 2, or missing. We first ignored missing data and estimated the reference allele frequency among nonmissing genotypes, or 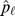. Following the *smartpca* program [12], we standardized the nonmissing entries in 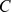 by
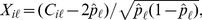
(1)where 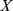 is a matrix with the same dimensions as 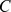. If a locus was monomorphic in a dataset (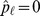 or 1), eq. 1 is undefined, and we set all entries in the column of 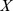 for this locus to zero. Entries representing missing data were set to zero in 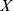 as well.

We performed PCA by applying the function *eigen* in *R* (www.r-project.org) to the 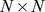 matrix 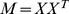 [43]. The coordinates of the 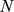 individuals on the 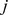
th PC are given by 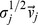, where 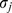 is the 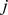
th eigenvalue of 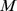, sorted in decreasing order, and 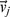 is the corresponding eigenvector. The proportion of variance explained by the 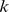
th PC is calculated as 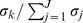, where 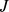 is the total number of eigenvectors of 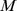. This quantity measures the variation among individuals along the 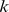
th PC direction, relative to the total variance in the standardized genotypic matrix 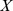. In our examples, 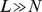, and 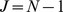 because 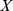 has rank 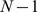 after standardization (eq. 1).

We note that some studies have used the eigenvectors 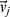 directly as PCs, so that all PCs have equal variance. We follow an alternative convention [43], [53], reporting PCs using 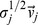, so that the proportions of variance explained by each PC are reflected on the PCA plot. In PCA plots superimposed on geographic maps, because horizontal and vertical axes are plotted on different scales, PC1 and PC2 can appear to not be perpendicular.

### Procrustes analysis and permutation test

We applied Procrustes analysis [13], [15] to compare the individual-level coordinates of the first two components (PC1 and PC2) in the PCA performed on the SNP data to the geographic coordinates. Procrustes analysis minimizes the sum of squared Euclidean distances between two sets of points (two “maps”) by transforming one set of points to optimally match the other set, while preserving the relative pairwise distances among all points within maps. Possible transformations include translation, scaling, rotation, and reflection. The similarity between two maps is then quantified by a Procrustes similarity statistic 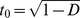, in which 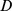 is the minimum sum of squared Euclidean distances between the two maps across all possible transformations. 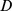, which is given by equation 6 in Wang *et al.* [15], has been scaled to have minimum 0 and maximum 1. The similarity statistic 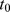 therefore also ranges from 0 to 1. In our analyses, we fixed the geographic coordinates and Procrustes-transformed the PCA coordinates in order to superimpose the PCA maps on the geographic maps. In addition to 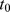, we also report the rotation angle 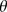 of the PCA map as given by the Procrustes analysis, measured in degrees counterclockwise.

To test the statistical significance of 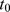, we used a permutation test. In each permutation, we randomly permuted the population geographic locations, assigning all individuals from the same population to a single geographic location in the permuted dataset. We then applied Procrustes analysis to compute the similarity score 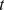 between the PCA coordinates and the randomly permuted geographic coordinates. We calculated the 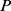-value as 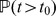, representing the probability of observing a similarity statistic higher than 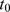 under the null hypothesis that no geographic pattern exists in the population structure. For each dataset, we employed 100,000 permutations for the permutation test.

### Analyses with populations excluded individually

We investigated the effect of each population on our PCA and Procrustes analysis using a leave-one-out approach. For each dataset, we excluded one population at a time and repeated PCA to obtain a new set of genetic coordinates (for each population excluded, this PCA started from the same final set of individuals after exclusions owing to missing data and PCA outliers, and we did not repeat the search for outliers). We then performed two Procrustes analyses. In the first one, we compared the new PCA coordinates and the original PCA coordinates obtained before removing any population. This comparison was based on the common set of individuals included in both analyses, and its similarity score was denoted 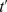. In the second Procrustes analysis, we computed the similarity between the new set of PCA coordinates and the corresponding geographic coordinates, denoting the similarity score by 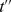.

### Subsets of loci

To investigate the effect of the number of markers on our results, we created a series of marker lists by randomly selecting 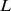 loci from the 32,991 total loci. These marker lists were selected independently of each other and had 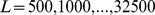. We then repeated PCA and Procrustes analysis for each geographic region using genotypes at the loci in each of our marker lists. For Sub-Saharan Africa, we used the dataset that excludes hunter-gatherer populations and the Mbororo Fulani. Given 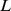, the analyses for different geographic regions are based on the same set of markers, so that their results are comparable.

### 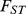 estimation

We calculated 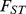 in each dataset using Weir and Cockerham's estimator (eq. 10 in [54]) based on all 32,991 loci.

## Supporting Information

Figure S1Procrustes analysis of genetic and geographic coordinates of European populations, when reducing the maximal sample size to 50. That is, for each population that has sample size 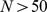 in Figure 2, we reduce the sample size to 50 by randomly excluding 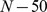 individuals. (A) Geographic coordinates of 37 populations. (B) Procrustes-transformed PCA plot of genetic variation. The Procrustes analysis is based on the unprojected latitude-longitude coordinates and PC1-PC2 coordinates of 721 individuals. PC1 and PC2 are indicated by dotted lines, crossing over the centroid of all individuals. Population abbreviations can be found in the caption of Figure 2. PC1 and PC2 account for 0.35% and 0.25% of the total variance, respectively. The Procrustes similarity is 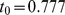 (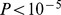). The rotation angle of the PCA map is 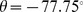. 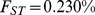.(PDF)

Figure S2Procrustes analysis of genetic and geographic coordinates of Sub-Saharan African populations, excluding Maasai (MKK) as well as Mbororo Fulani and four hunter-gatherer populations. (A) Geographic coordinates of 22 populations. (B) Procrustes-transformed PCA plot of genetic variation. The Procrustes analysis is based on the unprojected latitude-longitude coordinates and PC1-PC2 coordinates of 318 individuals. PC1 and PC2 are indicated by dotted lines, crossing over the centroid of all individuals. PC1 and PC2 account for 0.89% and 0.75% of the total variance, respectively. The Procrustes similarity statistic is 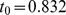 (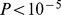). The rotation angle of the PCA map is 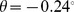.(PDF)

Figure S3 Procrustes analysis of genetic and geographic coordinates of Sub-Saharan African populations, including 23 populations in Figure 3 plus Mbororo Fulani and four hunter-gatherer populations (Biaka Pygmy, Mbuti Pygmy, !Kung, and San). (A) Geographic coordinates of all 28 populations. (B-G) Procrustes-transformed PCA plots of genetic variation. (B) All 28 populations. (C) 23 populations and Mbororo Fulani. (D) 23 populations and Biaka Pygmy. (E) 23 populations and Mbuti Pygmy. (F) 23 populations and !Kung. (G) 23 populations and San. Results are summarized in Table S7.
(PDF)

Figure S4Histograms of the Procrustes similarity 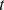 of 100,000 permutations for the Sub-Saharan African examples in Figure S3. The blue vertical lines indicate the value of 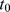. (A) All 28 populations (corresponding to Figure S3B, 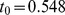, 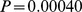). (B) 23 populations and Mbororo Fulani (Figure S3C, 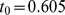, 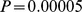). (C) 23 populations and Biaka Pygmy (Figure S3D, 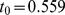, 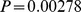). (D) 23 populations and Mbuti Pygmy (Figure S3E, 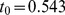, 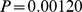). (E) 23 populations and !Kung (Figure S3F, 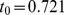, 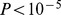). (F) 23 populations and San (Figure S3G, 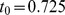, 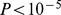).(PDF)

Figure S5Procrustes analysis of genetic and geographic coordinates of Asian populations, excluding Irula. (A) Geographic coordinates of 43 populations. (B) Procrustes-transformed PCA plot of genetic variation. The Procrustes analysis is based on the unprojected latitude-longitude coordinates and PC1-PC2 coordinates of 725 individuals. PC1 and PC2 are indicated by dotted lines, crossing over the centroid of all individuals. PC1 and PC2 account for 5.55% and 0.74% of the total variance, respectively. The Procrustes similarity statistic is 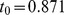 (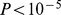). The rotation angle of the PCA map is 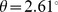.(PDF)

Figure S6Procrustes analysis of genetic and geographic coordinates of East Asian populations, excluding Tibetans. (A) Geographic coordinates of 22 populations. (B) Procrustes-transformed PCA plot of genetic variation. The Procrustes analysis is based on the unprojected latitude-longitude coordinates and PC1-PC2 coordinates of 303 individuals. PC1 and PC2 are indicated by dotted lines, crossing over the centroid of all individuals. PC1 and PC2 account for 1.72% and 1.02% of the total variance, respectively. The Procrustes similarity statistic is 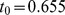 (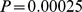). The rotation angle of the PCA map is 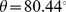.(PDF)

Figure S7 Data preparation procedure for creating datasets for different geographic regions.
(PDF)

Figure S8 Data-processing procedures for datasets from different geographic regions. (A) The worldwide dataset in Figure 1. (B) The European dataset in Figure 2. (C) The Sub-Saharan African dataset in Figure 3 (excluding Mbororo Fulani and four hunter-gatherer populations). (D) The Asian dataset in Figure 4. (E) The East Asian dataset in Figure 5. (F) The Central/South Asian dataset in Figure 6.
(PDF)

Figure S9 Data-processing procedure for the supplementary example of Sub-Saharan Africa when including Mbororo Fulani and four hunter-gatherer populations (Biaka Pygmy, Mbuti Pygmy, !Kung, and San). Similar procedures (not shown) were also used to prepare datasets for the analyses in Figure S3C-S3G, in each of which only one outlier population was included.
(PDF)

Table S1 Populations included in this study (Part I).
(PDF)

Table S2 Populations included in this study (Part II).
(PDF)

Table S3 Populations included in this study (Part III).
(PDF)

Table S4 Change of the Procrustes similarity when excluding one population from the worldwide example.
(PDF)

Table S5 Change of the Procrustes similarity when excluding one population from the European example.
(PDF)

Table S6 Change of the Procrustes similarity when excluding one population from the Sub-Saharan African example.
(PDF)

Table S7 Summary of the results for Sub-Saharan Africa when all or one of five additional African populations are included (corresponding to Figure S3).
(PDF)

Table S8 Change of the Procrustes similarity when excluding one population from the Asian example.
(PDF)

Table S9 Change of the Procrustes similarity when excluding one population from the East Asian example.
(PDF)

Table S10 Change of the Procrustes similarity when excluding one population from the Central/South Asian example.
(PDF)

Table S11 Samples identified as PCA outliers in the analyses for different geographic regions.
(PDF)

## References

[1] Sokal RR, Oden NL, Wilson C (1991) Genetic evidence for the spread of agriculture in Europe by demic diffusion. Nature 351: 143–145. 2030731 10.1038/351143a0

[2] Cavalli-Sforza LL, Menozzi P, Piazza A (1994) The History and Geography of Human Genes. Princeton: Princeton University Press.

[3] Barbujani G (2000) Geographic patterns: how to identify them and why. Hum Biol 72: 133–153. 10721615

[4] Cavalli-Sforza LL, Feldman MW (2003) The application of molecular genetic approaches to the study of human evolution. Nat Genet 33 Suppl:266–275. 12610536 10.1038/ng1113

[5] Novembre J, Ramachandran S (2011) Perspectives on human population structure at the cusp of the sequencing era. Annu Rev Genomics Hum Genet 12: 245–274. 21801023 10.1146/annurev-genom-090810-183123

[6] Ramachandran S, Deshpande O, Roseman CC, Rosenberg NA, Feldman MW, et al (2005) Support from the relationship of genetic and geographic distance in human populations for a serial founder effect originating in Africa. Proc Natl Acad Sci USA 102: 15942–15947. 16243969 10.1073/pnas.0507611102 PMC1276087

[7] Li JZ, Absher DM, Tang H, Southwick AM, Casto AM, et al (2008) Worldwide human relationships inferred from genome-wide patterns of variation. Science 319: 1100–1104. 18292342 10.1126/science.1153717

[8] Jakobsson M, Scholz SW, Scheet P, Gibbs JR, VanLiere JM, et al (2008) Genotype, haplotype and copy-number variation in worldwide human populations. Nature 451: 998–1003. 18288195 10.1038/nature06742

[9] Novembre J, Johnson T, Bryc K, Kutalik Z, Boyko AR, et al (2008) Genes mirror geography within Europe. Nature 456: 98–101. 18758442 10.1038/nature07331 PMC2735096

[10] Biswas S, Scheinfeldt LB, Akey JM (2009) Genome-wide insights into the patterns and determinants of fine-scale population structure in humans. Am J Hum Genet 84: 641–650. 19442770 10.1016/j.ajhg.2009.04.015 PMC2681007

[11] Menozzi P, Piazza A, Cavalli-Sforza L (1978) Synthetic maps of human gene frequencies in Europeans. Science 201: 786–792. 356262 10.1126/science.356262

[12] Patterson N, Price AL, Reich D (2006) Population structure and eigenanalysis. PLoS Genet 2: e190 doi:10.1371/journal.pgen.0020190. 17194218 10.1371/journal.pgen.0020190 PMC1713260

[13] Cox TF, Cox MAA (2001) Multidimensional Scaling. Boca Raton: Chapman & Hall, 2nd edition.

[14] Paschou P, Ziv E, Burchard EG, Choudhry S, Rodriguez-Cintron W, et al (2007) PCA-correlated SNPs for structure identification in worldwide human populations. PLoS Genet 3: e160 doi:10.1371/journal.pgen.0030160. 10.1371/journal.pgen.0030160 PMC1988848 17892327

[15] Wang C, Szpiech ZA, Degnan JH, Jakobsson M, Pemberton TJ, et al (2010) Comparing spatial maps of human population-genetic variation using Procrustes analysis. Stat Appl Genet Mol Biol 9: Article 13. 20196748 10.2202/1544-6115.1493 PMC2861313

[16] Lao O, Lu TT, Nothnagel M, Junge O, Freitag-Wolf S, et al (2008) Correlation between genetic and geographic structure in Europe. Curr Biol 18: 1241–1248. 18691889 10.1016/j.cub.2008.07.049

[17] Heath SC, Gut IG, Brennan P, McKay JD, Bencko V, et al (2008) Investigation of the fine structure of European populations with applications to disease association studies. Eur J Hum Genet 16: 1413–1429. 19020537 10.1038/ejhg.2008.210

[18] Jakkula E, Rehnström K, Varilo T, Pietiläinen OPH, Paunio T, et al (2008) The genome-wide patterns of variation expose significant substructure in a founder population. Am J Hum Genet 83: 787–794. 19061986 10.1016/j.ajhg.2008.11.005 PMC2668058

[19] Hoggart CJ, O'Reilly PF, Kaakinen M, Zhang W, Chambers JC, et al (2012) Fine-scale estimation of location of birth from genome-wide single-nucleotide polymorphism data. Genetics 190: 669–677. 22095078 10.1534/genetics.111.135657 PMC3276643

[20] Price AL, Helgason A, Palsson S, Stefansson H, St Clair D, et al (2009) The impact of divergence time on the nature of population structure: an example from Iceland. PLoS Genet 5: e1000505 doi:10.1371/journal.pgen.1000505. 19503599 10.1371/journal.pgen.1000505 PMC2684636

[21] Salmela E, Lappalainen T, Liu J, Sistonen P, Andersen PM, et al (2011) Swedish population substructure revealed by genome-wide single nucleotide polymorphism data. PLoS ONE 6: e16747 doi:10.1371/journal.pone.0016747. 21347369 10.1371/journal.pone.0016747 PMC3036708

[22] Xing J, Watkins WS, Witherspoon DJ, Zhang Y, Guthery SL, et al (2009) Fine-scaled human genetic structure revealed by SNP microarrays. Genome Res 19: 815–825. 19411602 10.1101/gr.085589.108 PMC2675970

[23] Xing J, Watkins WS, Shlien A, Walker E, Huff CD, et al (2010) Toward a more uniform sampling of human genetic diversity: a survey of worldwide populations by high-density genotyping. Genomics 96: 199–210. 20643205 10.1016/j.ygeno.2010.07.004 PMC2945611

[24] The HUGO Pan-Asian SNP Consortium (2009) Mapping human genetic diversity in Asia. Science 326: 1541–1545. 20007900 10.1126/science.1177074

[25] Tian C, Kosoy R, Lee A, Ransom M, Belmont JW, et al (2008) Analysis of East Asia genetic substructure using genome-wide SNP arrays. PLoS ONE 3: e3862 doi:10.1371/journal.pone.0003862. 19057645 10.1371/journal.pone.0003862 PMC2587696

[26] Bryc K, Auton A, Nelson MR, Oksenberg JR, Hauser SL, et al (2010) Genome-wide patterns of population structure and admixture in West Africans and African Americans. Proc Natl Acad Sci USA 107: 786–791. 20080753 10.1073/pnas.0909559107 PMC2818934

[27] Sikora M, Laayouni H, Calafell F, Comas D, Bertranpetit J (2011) A genomic analysis identifies a novel component in the genetic structure of sub-Saharan African populations. Eur J Hum Genet 19: 84–88. 20736976 10.1038/ejhg.2010.141 PMC3039508

[28] Chen J, Zheng H, Bei JX, Sun L, Jia WH, et al (2009) Genetic structure of the Han Chinese population revealed by genome-wide SNP variation. Am J Hum Genet 85: 775–785. 19944401 10.1016/j.ajhg.2009.10.016 PMC2790583

[29] Xu S, Yin X, Li S, Jin W, Lou H, et al (2009) Genomic dissection of population substructure of Han Chinese and its implication in association studies. Am J Hum Genet 85: 762–774. 19944404 10.1016/j.ajhg.2009.10.015 PMC2790582

[30] Yamaguchi-Kabata Y, Nakazono K, Takahashi A, Saito S, Hosono N, et al (2008) Japanese population structure, based on SNP genotypes from 7003 individuals compared to other ethnic groups: effects on population-based association studies. Am J Hum Genet 83: 445–456. 18817904 10.1016/j.ajhg.2008.08.019 PMC2561928

[31] Pemberton TJ, Absher D, Feldman MW, Myers RM, Rosenberg NA, et al Genomic patterns of homozygosity in worldwide human populations. Am J Hum Genet (in press). 10.1016/j.ajhg.2012.06.014 PMC3415543 22883143

[32] Simonson T, Yang Y, Huff CD, Yun H, Qin G, et al (2010) Genetic evidence for high-altitude adaptation in Tibet. Science 329: 72–75. 20466884 10.1126/science.1189406

[33] The International HapMap 3 Consortium (2010) Integrating common and rare genetic variation in diverse human populations. Nature 467: 52–58. 20811451 10.1038/nature09298 PMC3173859

[34] Auton A, Bryc K, Boyko AR, Lohmueller KE, Novembre J, et al (2009) Global distribution of genomic diversity underscores rich complex history of continental human populations. Genome Res 19: 795–803. 19218534 10.1101/gr.088898.108 PMC2675968

[35] Bowcock AM, Ruiz-Linares A, Tomfohrde J, Minch E, Kidd JR, et al (1994) High resolution of human evolutionary trees with polymorphic microsatellites. Nature 368: 455–457. 7510853 10.1038/368455a0

[36] Rosenberg NA, Pritchard JK, Weber JL, Cann HM, Kidd KK, et al (2002) Genetic structure of human populations. Science 298: 2381–2385. 12493913 10.1126/science.1078311

[37] Tishkoff SA, Reed FA, Friedlaender FR, Ehret C, Ranciaro A, et al (2009) The genetic structure and history of Africans and African Americans. Science 324: 1035–1044. 19407144 10.1126/science.1172257 PMC2947357

[38] Henn BM, Gignoux CR, Jobin M, Granka JM, Macpherson JM, et al (2011) Hunter-gatherer genomic diversity suggests a southern African origin for modern humans. Proc Natl Acad Sci USA 108: 5154–5162. 21383195 10.1073/pnas.1017511108 PMC3069156

[39] Bregel Y (2003) An Historical Atlas of Central Asia. Boston: Brill.

[40] Du R, Yip VF (1993) Ethnic Groups in China. Beijing: Science Press.

[41] Powell GT, Yang H, Tyler-Smith C, Xue Y (2007) The population history of the Xibe in northern China: a comparison of autosomal, mtDNA and Y-chromosomal analyses of migration and gene ow. Forensic Sci Int Genet 1: 115–119. 19083740 10.1016/j.fsigen.2007.01.015

[42] Weir BS (1996) Genetic Data Analysis II. Sunderland, MA: Sinauer.

[43] McVean G (2009) A genealogical interpretation of principal components analysis. PLoS Genet 5: e1000686 doi:10.1371/journal.pgen.1000686. 19834557 10.1371/journal.pgen.1000686 PMC2757795

[44] Novembre J, Stephens M (2008) Interpreting principal component analyses of spatial population genetic variation. Nature Genet 40: 646–649. 18425127 10.1038/ng.139 PMC3989108

[45] Rosenberg NA (2011) A population-genetic perspective on the similarities and differences among worldwide human populations. Hum Biol 83: 659–684. 22276967 10.3378/027.083.0601 PMC3531797

[46] Engelhardt BE, Stephens M (2010) Analysis of population structure: a unifying framework and novel methods based on sparse factor analysis. PLoS Genet 6: e1001117 doi:10.1371/journal.pgen.1001117. 20862358 10.1371/journal.pgen.1001117 PMC2940725

[47] Rosenberg NA, Mahajan S, Ramachandran S, Zhao C, Pritchard JK, et al (2005) Clines, clusters, and the effect of study design on the inference of human population structure. PLoS Genet 1 doi:10.1371/journal.pgen.0010070. 10.1371/journal.pgen.0010070 PMC1310579 16355252

[48] Yang WY, Novembre J, Eskin E, Halperin E (2012) A model-based approach for analysis of spatial structure in genetic data. Nat Genet 44: 725–731. 22610118 10.1038/ng.2285 PMC3592563

[49] Pemberton TJ, Wang C, Li JZ, Rosenberg NA (2010) Inference of unexpected genetic relatedness among individuals in HapMap Phase III. Am J Hum Genet 87: 457–464. 20869033 10.1016/j.ajhg.2010.08.014 PMC2948801

[50] Nelson MR, Bryc K, King KS, Indap A, Boyko AR, et al (2008) The Population Reference Sample, POPRES: a resource for population, disease, and pharmacological genetics research. Am J Hum Genet 83: 347–358. 18760391 10.1016/j.ajhg.2008.08.005 PMC2556436

[51] Mailman MD, Feolo M, Jin Y, Kimura M, Tryka K, et al (2007) The NCBI dbGaP database of genotypes and phenotypes. Nat Genet 39: 1181–1186. 17898773 10.1038/ng1007-1181 PMC2031016

[52] Price AL, Patterson NJ, Plenge RM, Weinblatt ME, Shadick NA, et al (2006) Principal components analysis corrects for stratification in genome-wide association studies. Nature Genet 38: 904–909. 16862161 10.1038/ng1847

[53] Hastie T, Tibshirani R, Friedman J (2009) The Elements of Statistical Learning: Data Mining, Inference, and Prediction. New York: Springer, 2nd edition.

[54] Weir BS, Cockerham CC (1984) Estimating F-statistics for the analysis of population structure. Evolution 38: 1358–1370. 10.1111/j.1558-5646.1984.tb05657.x 28563791

