# Supplementary figures and images for: A Quantitative Comparison of the Similarity between Genes and Geography in Worldwide Human Populations
Source: PLoS Genet. 2012 Aug 23;8(8):e1002886. doi: 10.1371/journal.pgen.1002886 (PMC3426559; doi:10.1371/journal.pgen.1002886)

A

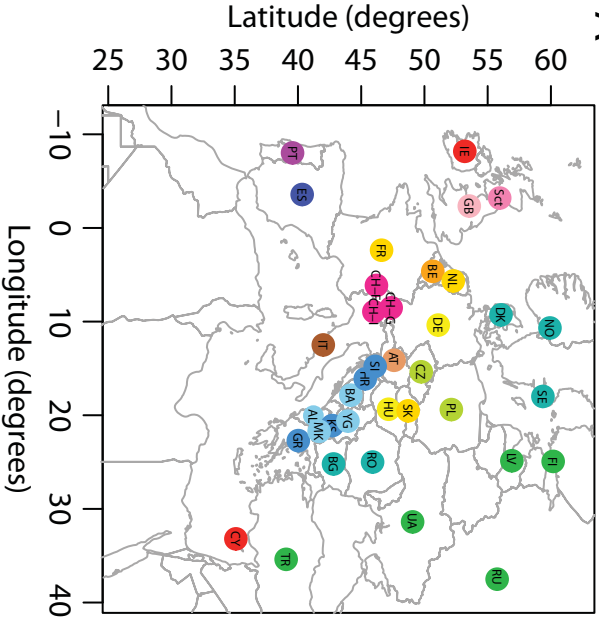

B

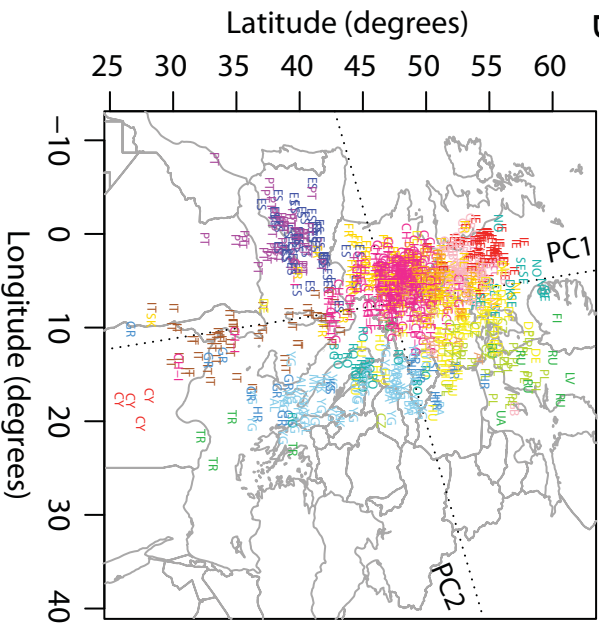

Supplement: Figure S1 — Procrustes analysis of genetic and geographic coordinates of European populations, when reducing the maximal sample size to 50. That is, for each population that has sample size in Figure 2, we reduce the sample size to 50 by randomly excluding individuals. (A) Geographic coordinates of 37 populations. (B) Procrustes-transformed PCA plot of genetic variation. The Procrustes analysis is based on the unprojected latitude-longitude coordinates and PC1-PC2 coordinates of 721 individuals. PC1 and PC2 are indicated by dotted lines, crossing over the centroid of all individuals. Population abbreviations can be found in the caption of Figure 2. PC1 and PC2 account for 0.35% and 0.25% of the total variance, respectively. The Procrustes similarity is (). The rotation angle of the PCA map is . . (PDF) [file pgen.1002886.s001.pdf]

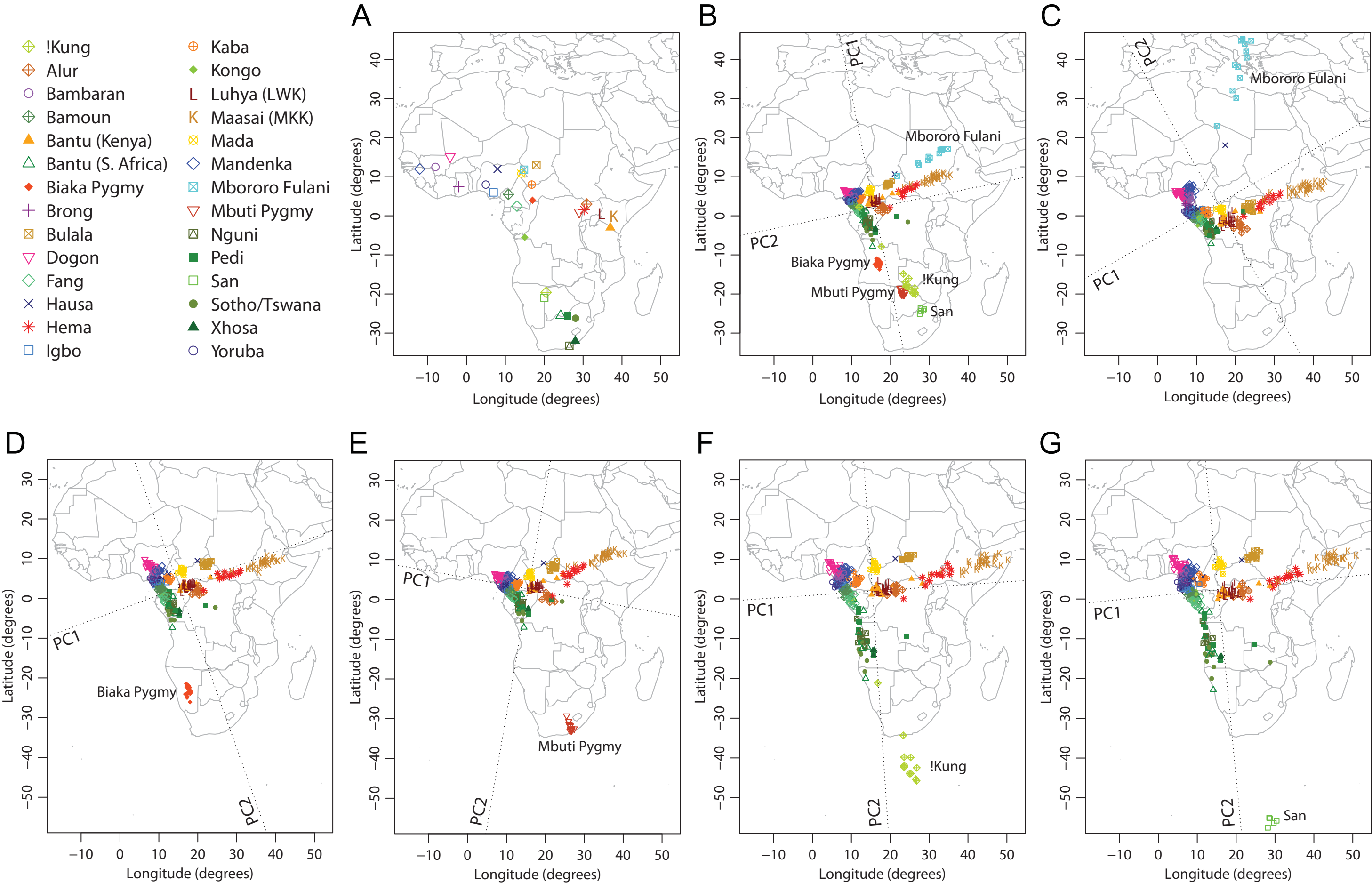

Supplement: Figure S3 — Procrustes analysis of genetic and geographic coordinates of Sub-Saharan African populations, including 23 populations in Figure 3 plus Mbororo Fulani and four hunter-gatherer populations (Biaka Pygmy, Mbuti Pygmy, !Kung, and San). (A) Geographic coordinates of all 28 populations. (B-G) Procrustes-transformed PCA plots of genetic variation. (B) All 28 populations. (C) 23 populations and Mbororo Fulani. (D) 23 populations and Biaka Pygmy. (E) 23 populations and Mbuti Pygmy. (F) 23 populations and !Kung. (G) 23 populations and San. Results are summarized in Table S7. (PDF) [file pgen.1002886.s003.pdf]

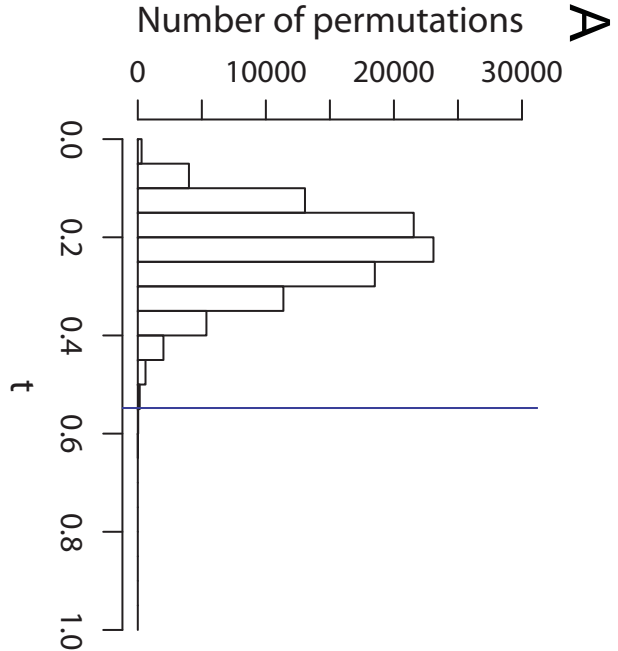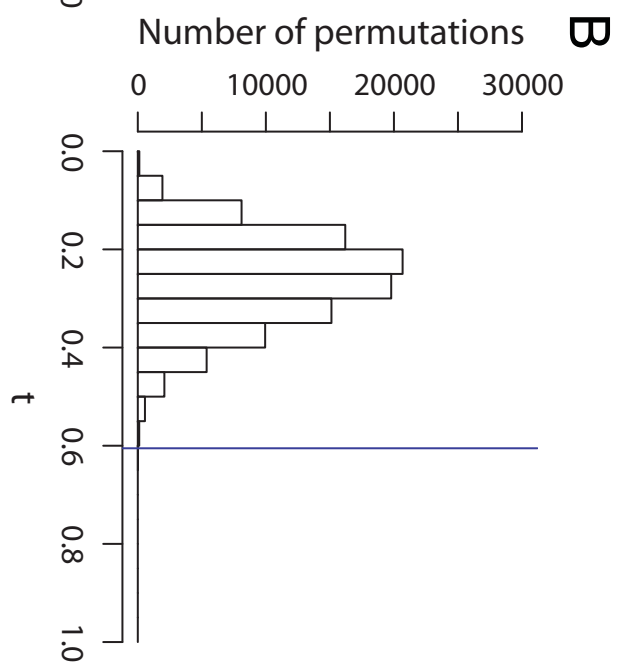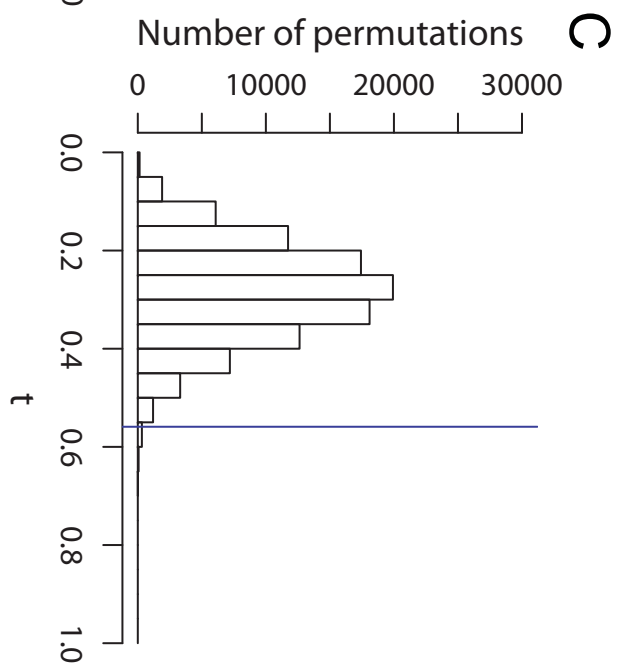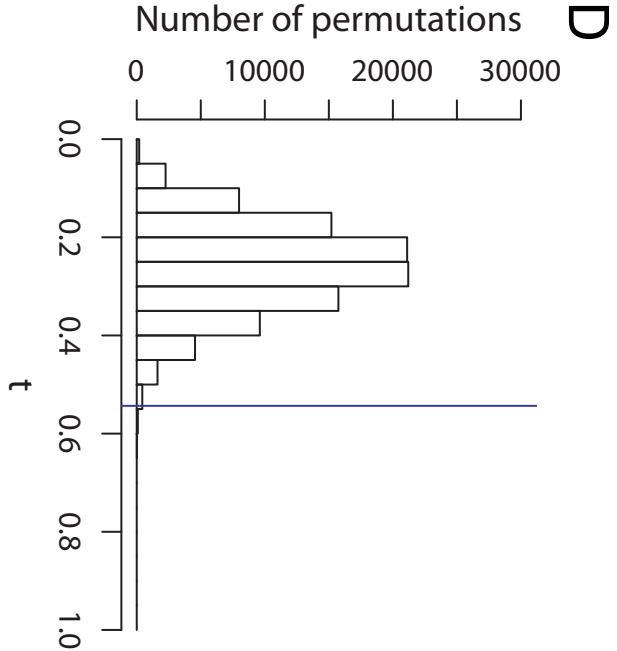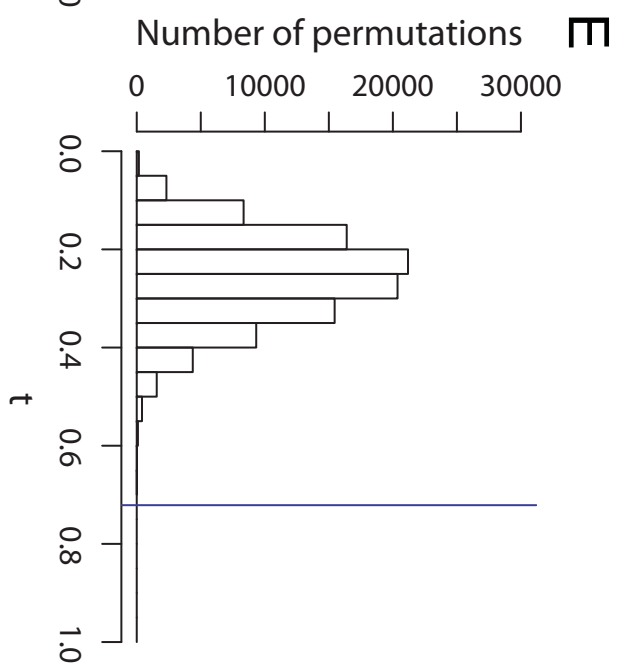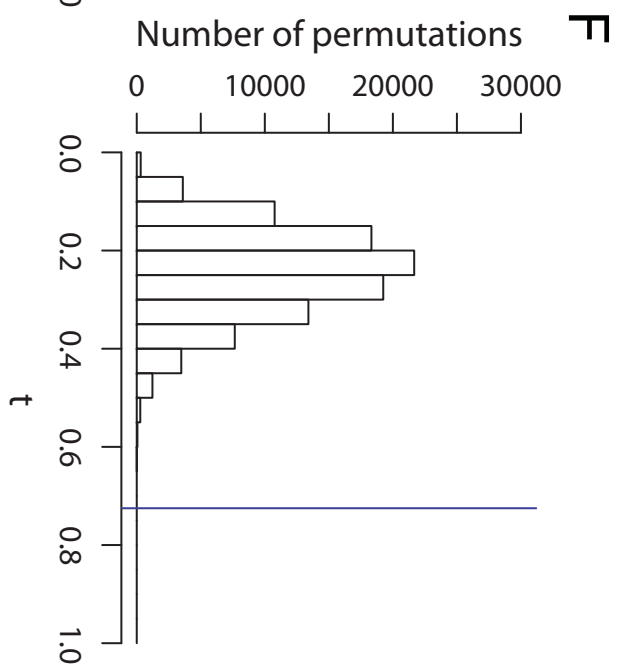

Supplement: Figure S4 — Histograms of the Procrustes similarity of 100,000 permutations for the Sub-Saharan African examples in Figure S3. The blue vertical lines indicate the value of . (A) All 28 populations (corresponding to Figure S3B, , ). (B) 23 populations and Mbororo Fulani (Figure S3C, , ). (C) 23 populations and Biaka Pygmy (Figure S3D, , ). (D) 23 populations and Mbuti Pygmy (Figure S3E, , ). (E) 23 populations and !Kung (Figure S3F, , ). (F) 23 populations and San (Figure S3G, , ). (PDF) [file pgen.1002886.s004.pdf]

A

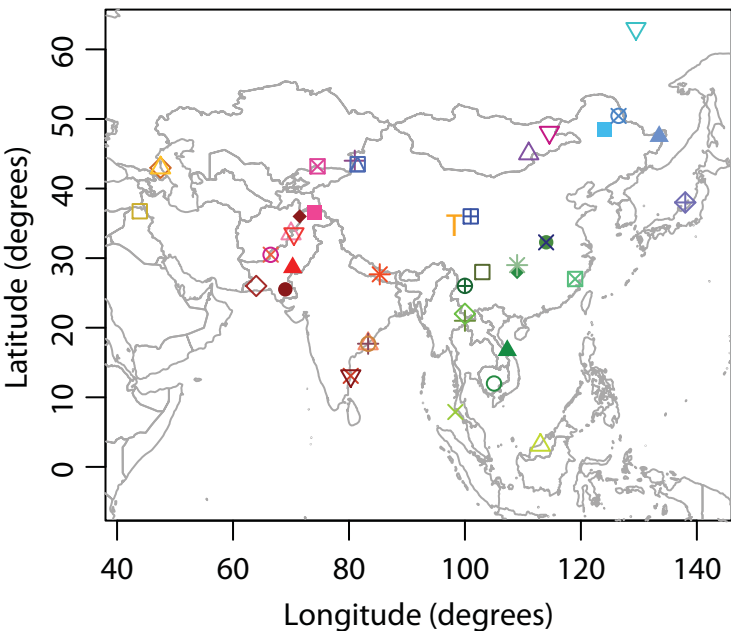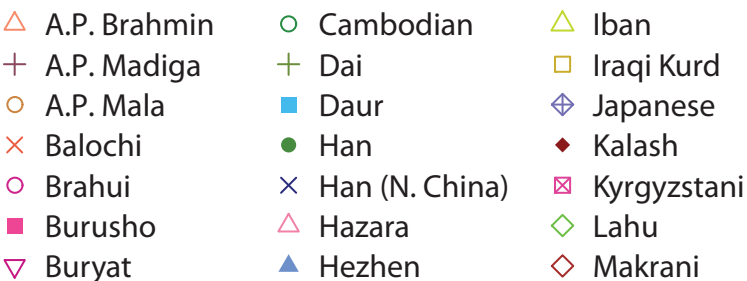

B

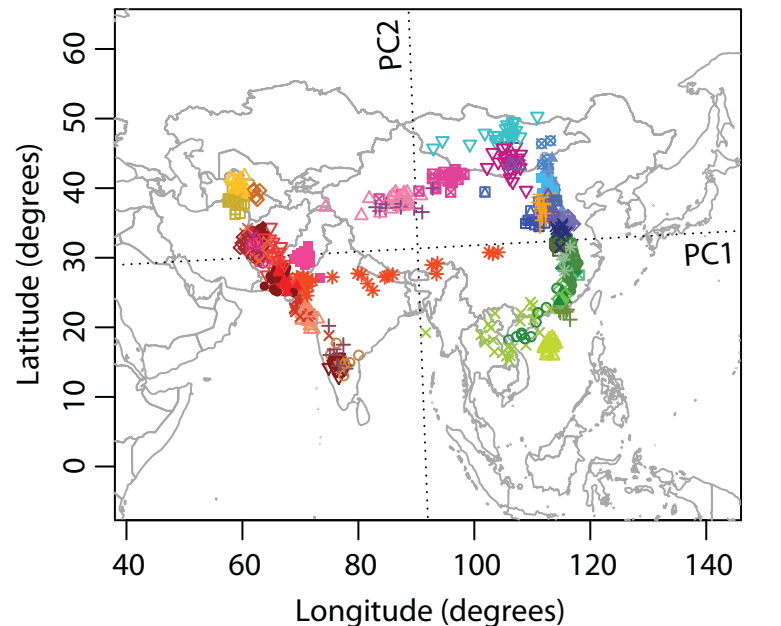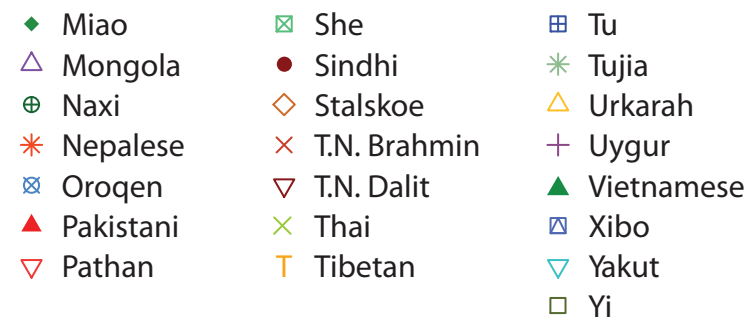

Supplement: Figure S5 — Procrustes analysis of genetic and geographic coordinates of Asian populations, excluding Irula. (A) Geographic coordinates of 43 populations. (B) Procrustes-transformed PCA plot of genetic variation. The Procrustes analysis is based on the unprojected latitude-longitude coordinates and PC1-PC2 coordinates of 725 individuals. PC1 and PC2 are indicated by dotted lines, crossing over the centroid of all individuals. PC1 and PC2 account for 5.55% and 0.74% of the total variance, respectively. The Procrustes similarity statistic is (). The rotation angle of the PCA map is . (PDF) [file pgen.1002886.s005.pdf]

A

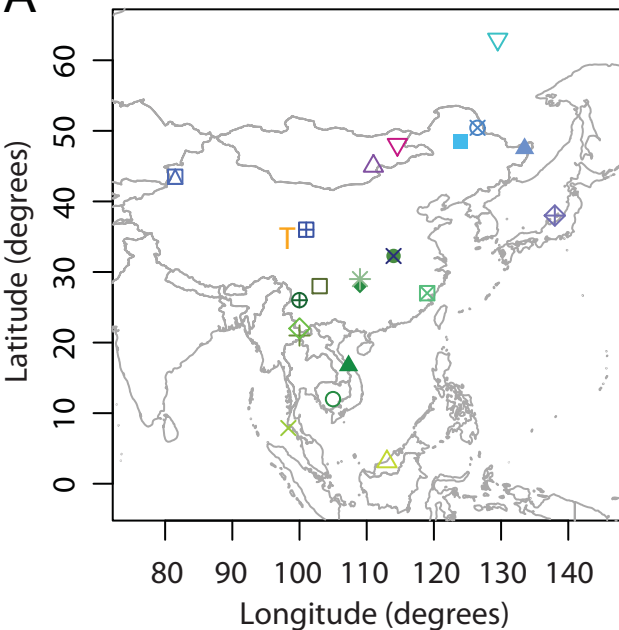

B

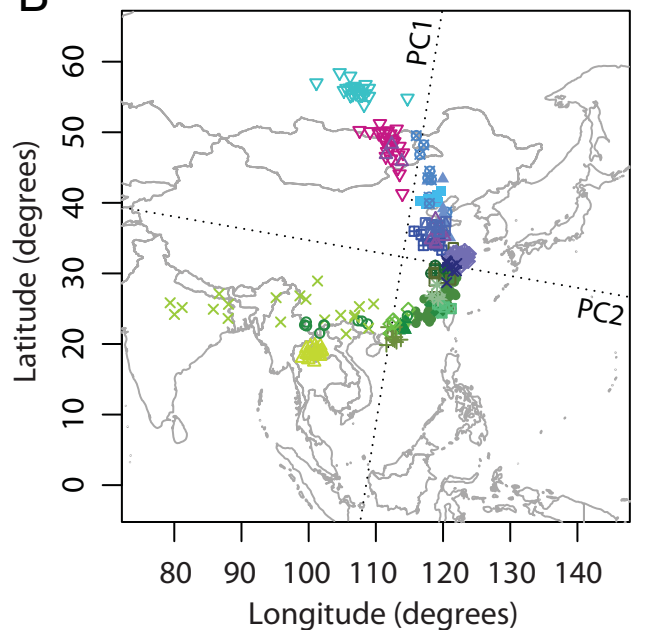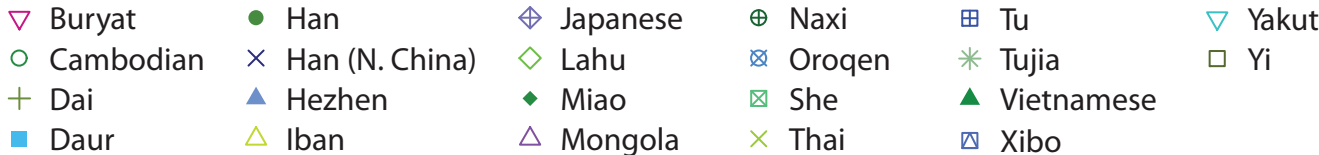

Supplement: Figure S6 — Procrustes analysis of genetic and geographic coordinates of East Asian populations, excluding Tibetans. (A) Geographic coordinates of 22 populations. (B) Procrustes-transformed PCA plot of genetic variation. The Procrustes analysis is based on the unprojected latitude-longitude coordinates and PC1-PC2 coordinates of 303 individuals. PC1 and PC2 are indicated by dotted lines, crossing over the centroid of all individuals. PC1 and PC2 account for 1.72% and 1.02% of the total variance, respectively. The Procrustes similarity statistic is (). The rotation angle of the PCA map is . (PDF) [file pgen.1002886.s006.pdf]

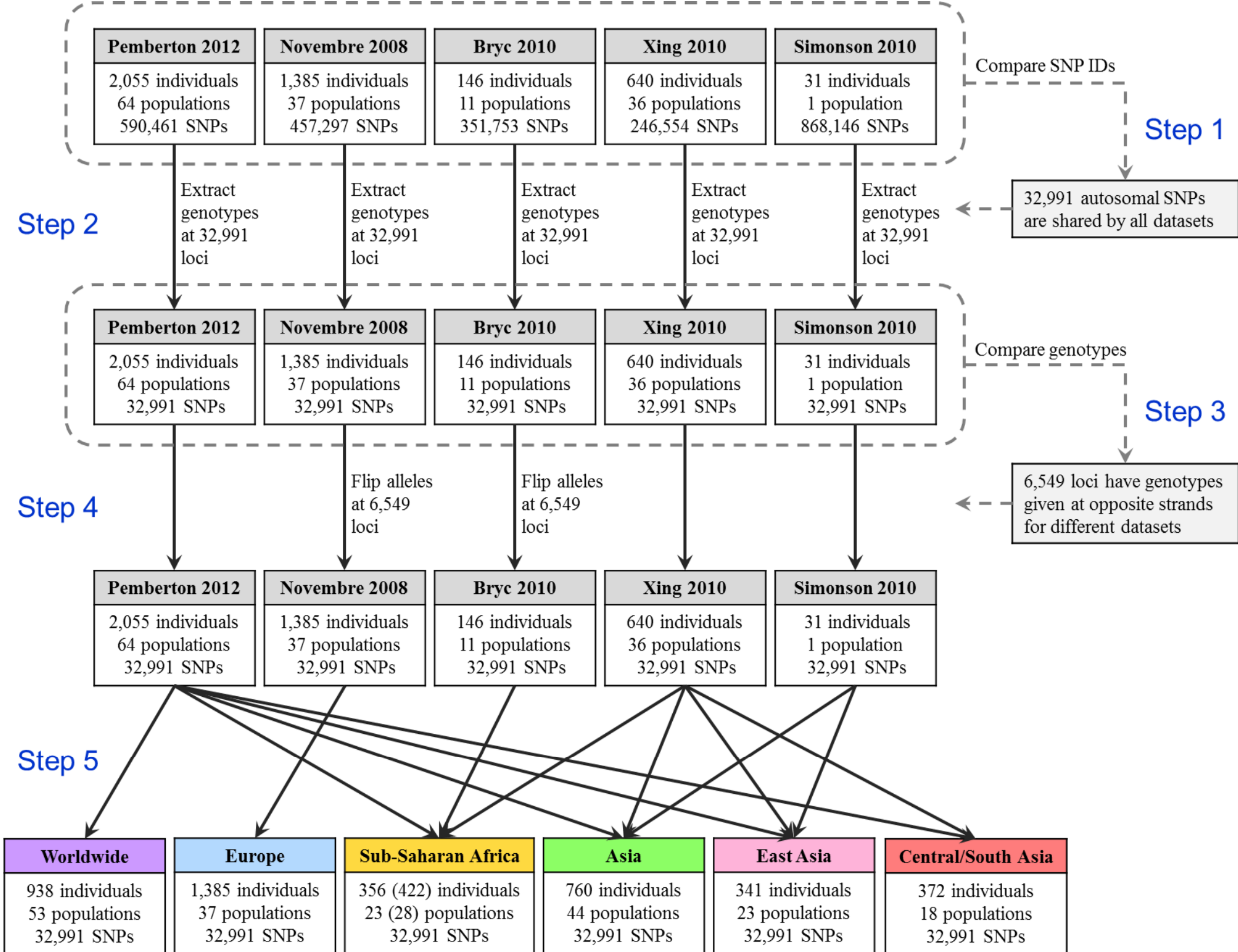

Supplement: Figure S7 — Data preparation procedure for creating datasets for different geographic regions. (PDF) [file pgen.1002886.s007.pdf]

A

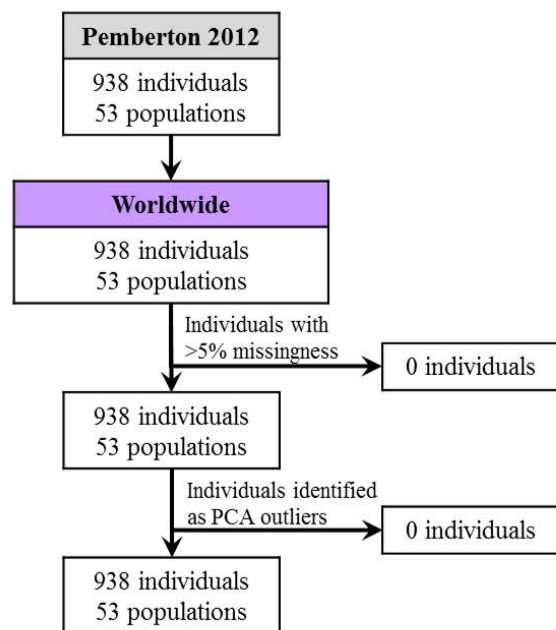

B

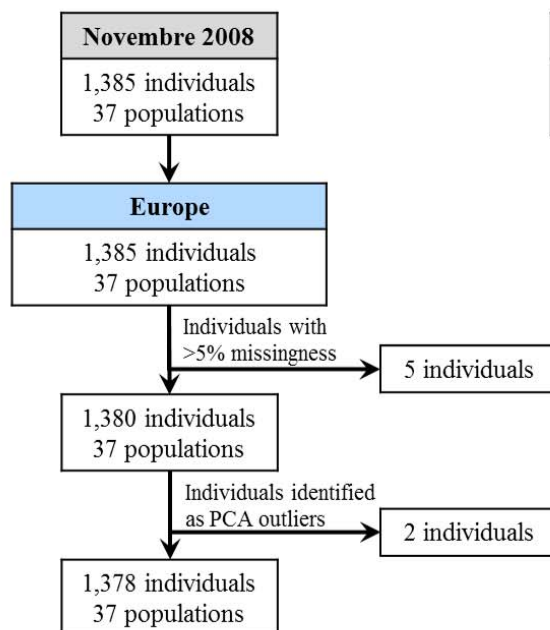

C

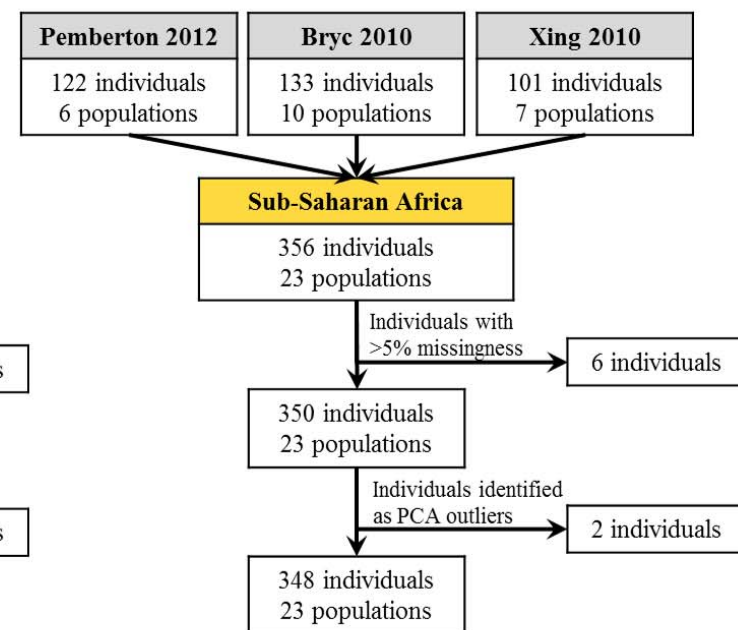

D

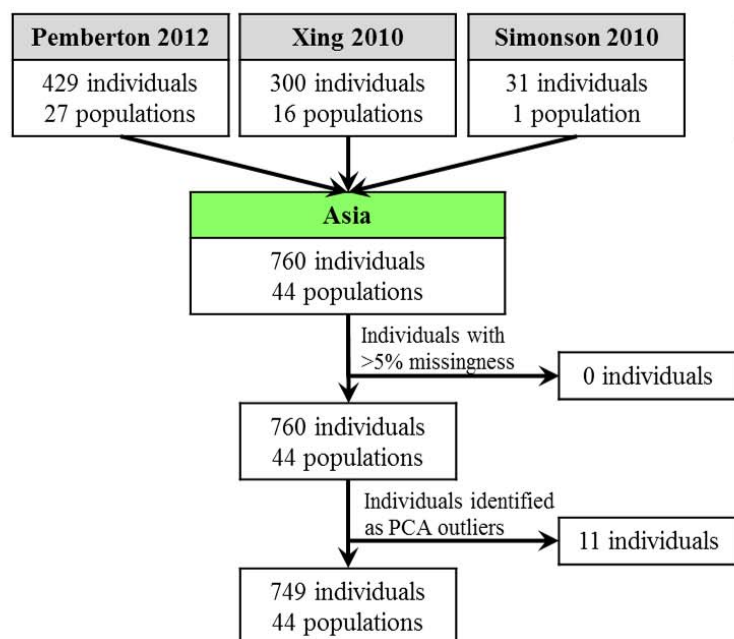

E

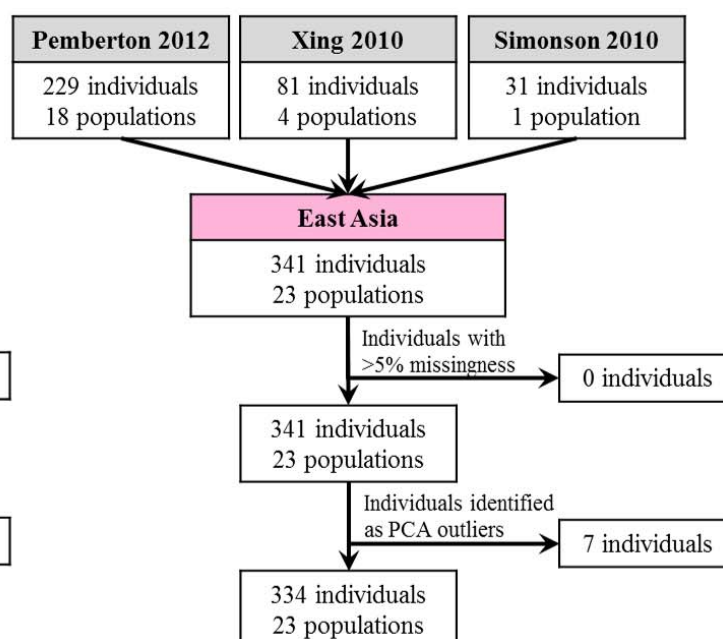

F

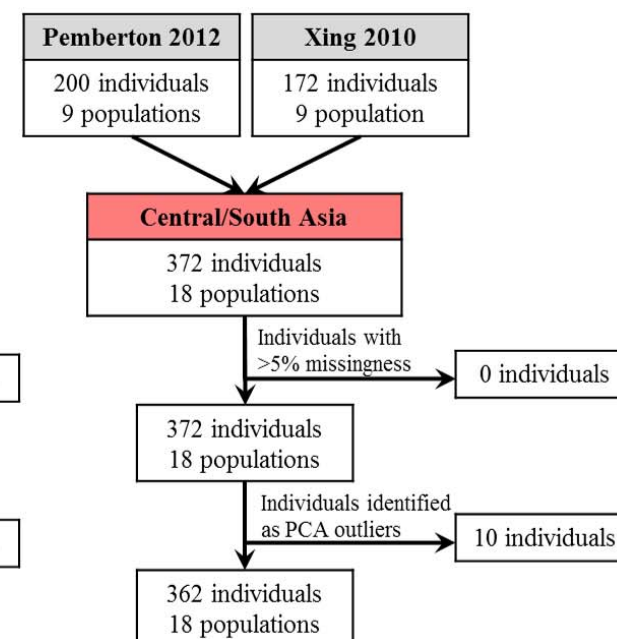

Supplement: Figure S8 — Data-processing procedures for datasets from different geographic regions. (A) The worldwide dataset in Figure 1. (B) The European dataset in Figure 2. (C) The Sub-Saharan African dataset in Figure 3 (excluding Mbororo Fulani and four hunter-gatherer populations). (D) The Asian dataset in Figure 4. (E) The East Asian dataset in Figure 5. (F) The Central/South Asian dataset in Figure 6. (PDF) [file pgen.1002886.s008.pdf]

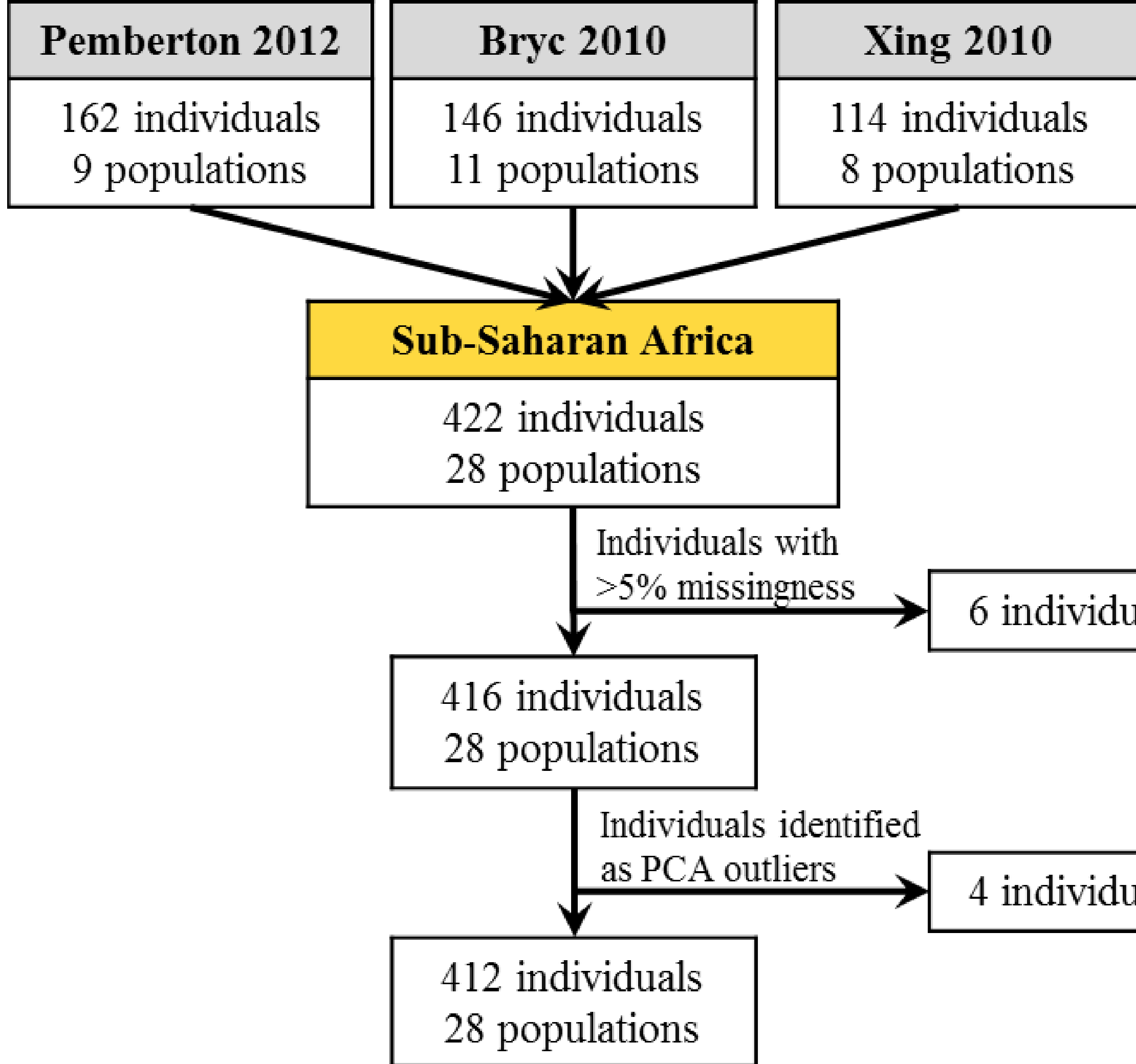

Supplement: Figure S9 — Data-processing procedure for the supplementary example of Sub-Saharan Africa when including Mbororo Fulani and four hunter-gatherer populations (Biaka Pygmy, Mbuti Pygmy, !Kung, and San). Similar procedures (not shown) were also used to prepare datasets for the analyses in Figure S3C-S3G, in each of which only one outlier population was included. (PDF) [file pgen.1002886.s009.pdf]
