# Supplementary material for: A Quantitative Comparison of the Similarity between Genes and Geography in Worldwide Human Populations
Source: PLoS Genet. 2012 Aug 23;8(8):e1002886. doi: 10.1371/journal.pgen.1002886 (PMC3426559; doi:10.1371/journal.pgen.1002886)
Supplement: Figure S2 — Procrustes analysis of genetic and geographic coordinates of Sub-Saharan African populations, excluding Maasai (MKK) as well as Mbororo Fulani and four hunter-gatherer populations. (A) Geographic coordinates of 22 populations. (B) Procrustes-transformed PCA plot of genetic variation. The Procrustes analysis is based on the unprojected latitude-longitude coordinates and PC1-PC2 coordinates of 318 individuals. PC1 and PC2 are indicated by dotted lines, crossing over the centroid of all individuals. PC1 and PC2 account for 0.89% and 0.75% of the total variance, respectively. The Procrustes similarity statistic is (). The rotation angle of the PCA map is . (PDF) [file pgen.1002886.s002.pdf]

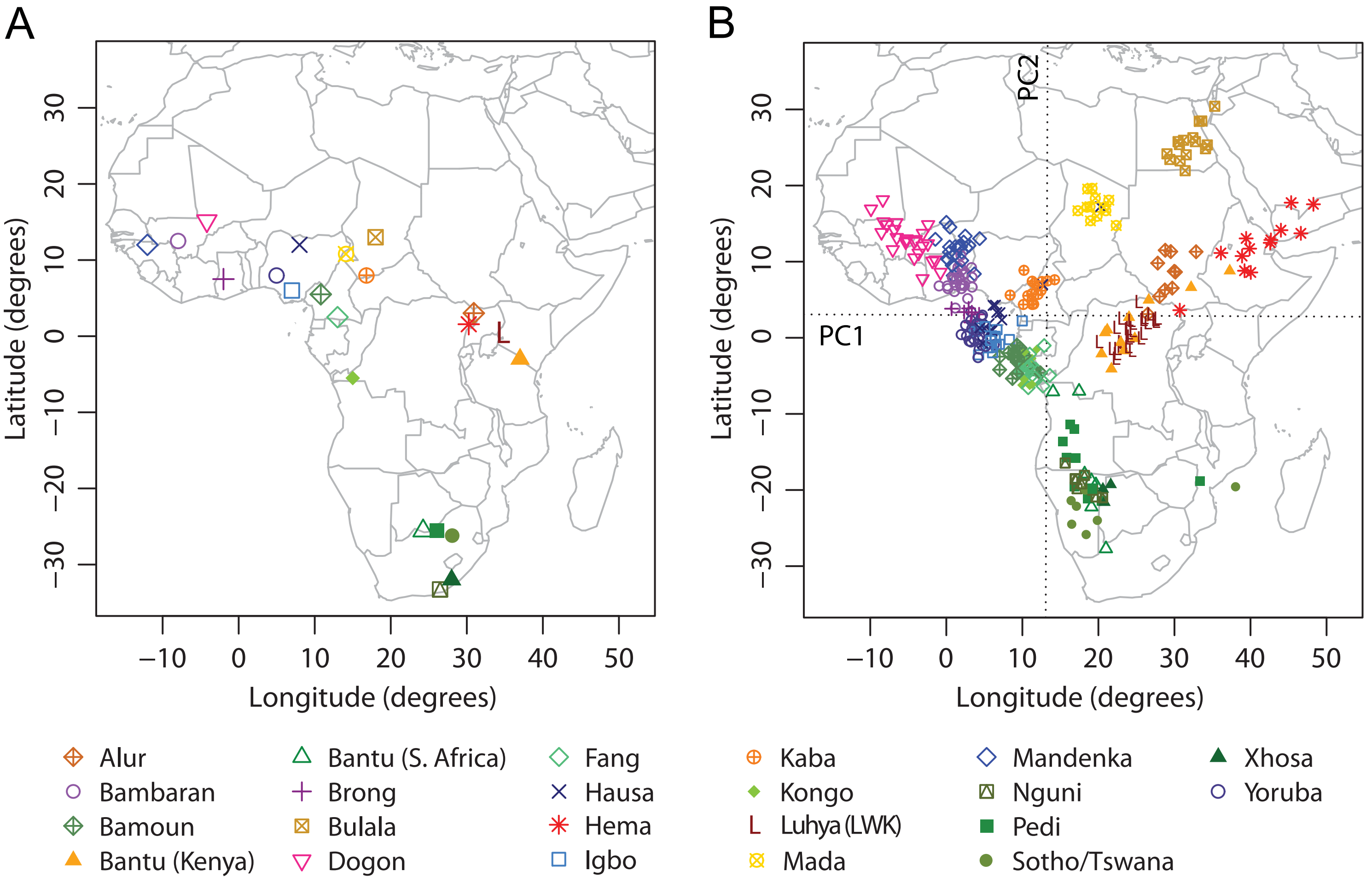
A
B
Latitude (degrees)
Longitude (degrees)
30
20
10
0
−10
−20
−30
−10
0
10
20
30
40
50
PC1
PC2
Alur
Bambaran
Bamoun
Bantu (Kenya)
Bantu (S. Africa)
Brong
Bulala
Dogon
Fang
Hausa
Hema
Igbo
Kaba
Kongo
Luhya (LWK)
Mada
Mandenka
Nguni
Pedi
Sotho/Tswana
Xhosa
Yoruba
