# Supplementary material for: A Quantitative Comparison of the Similarity between Genes and Geography in Worldwide Human Populations
Source: PLoS Genet. 2012 Aug 23;8(8):e1002886. doi: 10.1371/journal.pgen.1002886 (PMC3426559; doi:10.1371/journal.pgen.1002886)
Supplement: Table S1 — Populations included in this study (Part I). (PDF) [file pgen.1002886.s010.pdf]

Population	Latitude (degrees)	Longitude (degrees)	Source of coordinates	Sample size	High-missing-data samples	Genotyping platform	Source of SNP data	Datasets in which the population is included					
								World	Europe	Africa	Asia	E. Asia	C.S. Asia
Adygei	44	39	[45]	17	0	Illumina 650K	[31]	X					
Balochi	30.5	66.5	[45]	24	0	Illumina 650K	[31]	X			X		
Bantu (Kenya)	-3	37	[45]	11	0	Illumina 650K	[31]	X		X			
Bantu (S. Africa)	-25.6	24.3	[45]	8	0	Illumina 650K	[31]	X		X			
Basque	43	0	[45]	24	0	Illumina 650K	[31]	X					
Bedouin	31	35	[45]	45	0	Illumina 650K	[31]	X					
Biaka Pygmy	4	17	[45]	22	0	Illumina 650K	[31]	X		X			
Brahui	30.5	66.5	[45]	25	0	Illumina 650K	[31]	X			X		
Burusho	36.5	74	[45]	25	0	Illumina 650K	[31]	X			X		
Cambodian	12	105	[45]	10	0	Illumina 650K	[31]	X			X	X	
Colombian	3	-68	[45]	7	0	Illumina 650K	[31]	X					
Dai	21	100	[45]	10	0	Illumina 650K	[31]	X			X	X	
Daur	48.5	124	[45]	9	0	Illumina 650K	[31]	X			X	X	
Druze	32	35	[45]	42	0	Illumina 650K	[31]	X					
French	46	2	[45]	28	0	Illumina 650K	[31]	X					
Han	32.3	114	[45]	34	0	Illumina 650K	[31]	X			X	X	
Han (N. China)	32.3	114	[45]	10	0	Illumina 650K	[31]	X			X	X	
Hazara	33.5	70	[45]	22	0	Illumina 650K	[31]	X			X		X
Hezhen	47.5	133.5	[45]	9	0	Illumina 650K	[31]	X			X	X	
Italian	46	10	[45]	12	0	Illumina 650K	[31]	X					
Japanese	38	138	[45]	28	0	Illumina 650K	[31]	X			X	X	
Kalash	36.0	71.5	[45]	23	0	Illumina 650K	[31]	X			X		X
Karitiana	-10	-63	[45]	13	0	Illumina 650K	[31]	X					
Lahu	22	100	[45]	8	0	Illumina 650K	[31]	X			X	X	
Makrani	26	64	[45]	25	0	Illumina 650K	[31]	X			X		X
Mandenka	12	-12	[45]	22	0	Illumina 650K	[31]	X		X			
Maya	19	-91	[45]	21	0	Illumina 650K	[31]	X					
Mbuti Pygmy	1	29	[45]	13	0	Illumina 650K	[31]	X		X			
Melanesian	-6	155	[45]	11	0	Illumina 650K	[31]	X					
Miao	28	109	[45]	10	0	Illumina 650K	[31]	X			X	X	
Mongola	45	111	[45]	10	0	Illumina 650K	[31]	X			X	X	
Mozabite	32	3	[45]	27	0	Illumina 650K	[31]	X					
Naxi	26	100	[45]	8	0	Illumina 650K	[31]	X			X	X	
Ocadian	59	-3	[45]	15	0	Illumina 650K	[31]	X					
Oroqen	50.4	126.5	[45]	9	0	Illumina 650K	[31]	X			X	X	
Palestinian	32	35	[45]	46	0	Illumina 650K	[31]	X					
Papuan	-4	143	[45]	17	0	Illumina 650K	[31]	X					
Pathan	33.5	70.5	[45]	22	0	Illumina 650K	[31]	X			X		X
Pima	29	-108	[45]	14	0	Illumina 650K	[31]	X					
Russian	61	40	[45]	25	0	Illumina 650K	[31]	X					
San	-21	20	[45]	5	0	Illumina 650K	[31]	X		X			
Sardinian	40	9	[45]	28	0	Illumina 650K	[31]	X					
She	27	119	[45]	10	0	Illumina 650K	[31]	X			X	X	
Sindhi	25.5	69	[45]	24	0	Illumina 650K	[31]	X			X		X
Surui	-11	-62	[45]	8	0	Illumina 650K	[31]	X					

Table S1: Populations included in this study (Part I).
