# Supplementary material for: A Quantitative Comparison of the Similarity between Genes and Geography in Worldwide Human Populations
Source: PLoS Genet. 2012 Aug 23;8(8):e1002886. doi: 10.1371/journal.pgen.1002886 (PMC3426559; doi:10.1371/journal.pgen.1002886)
Supplement: Table S2 — Populations included in this study (Part II). (PDF) [file pgen.1002886.s011.pdf]

Population	Latitude (degrees)	Longitude (degrees)	Source of coordinates	Sample size	High-missing-data samples	Genotyping platform	Source of SNP data	Datasets in which the population is included					
								World	Europe	Africa	Asia	E. Asia	C.S. Asia
Tu	36	101	[45]	10	0	Illumina 650K	[31]	X			X	X	
Tujia	29	109	[45]	10	0	Illumina 650K	[31]	X			X	X	
Tuscan	43	11	[45]	7	0	Illumina 650K	[31]	X					
Uygur	44	81	[45]	10	0	Illumina 650K	[31]	X			X		X
Xibo	43.5	81.5	[45]	9	0	Illumina 650K	[31]	X			X	X	
Yakut	63.0	129.5	[45]	25	0	Illumina 650K	[31]	X			X	X	
Yi	28	103	[45]	10	0	Illumina 650K	[31]	X			X	X	
Yoruba	8	5	[45]	21	0	Illumina 650K	[31]	X		X			
Luhya (LWK)	0.6	34.8	[33]	30	0	HapMap3 rel2	[31]			X			
Maasai (MKK)	0	37.9	[33]	30	0	HapMap3 rel2	[31]			X			
Albania (AL)	41.2	20.1	[9]	3	0	Affymetrix 500K	[9]		X				
Austria (AT)	47.6	14.1	[9]	14	0	Affymetrix 500K	[9]		X				
Bosnia-Herzegovina (BA)	44.2	17.9	[9]	9	0	Affymetrix 500K	[9]		X				
Belgium (BE)	50.7	4.61	[9]	43	1	Affymetrix 500K	[9]		X				
Bulgaria (BG)	42.8	25.2	[9]	2	0	Affymetrix 500K	[9]		X				
Swiss-French (CH-F)	46.2	6.15	[9]	125	0	Affymetrix 500K	[9]		X				
Swiss-German (CH-G)	47.4	8.55	[9]	84	2	Affymetrix 500K	[9]		X				
Swiss-Italian (CH-I)	46	8.95	[9]	13	0	Affymetrix 500K	[9]		X				
Cyprus (CY)	35.1	33.2	[9]	4	0	Affymetrix 500K	[9]		X				
Czech Republic (CZ)	49.7	15.4	[9]	11	0	Affymetrix 500K	[9]		X				
Germany (DE)	51.1	10.4	[9]	71	2	Affymetrix 500K	[9]		X				
Denmark (DK)	56.1	9.25	[9]	1	0	Affymetrix 500K	[9]		X				
Spain (ES)	40.3	-3.57	[9]	136	0	Affymetrix 500K	[9]		X				
Finland (FI)	60.2	24.9	[9]	1	0	Affymetrix 500K	[9]		X				
France (FR)	46.6	2.39	[9]	89	0	Affymetrix 500K	[9]		X				
United Kingdom (GB)	53.5	-2.33	[9]	200	1	Affymetrix 500K	[9]		X				
Greece (GR)	40	22.7	[9]	8	0	Affymetrix 500K	[9]		X				
Croatia (HR)	45.3	16.1	[9]	8	0	Affymetrix 500K	[9]		X				
Hungary (HU)	47.2	19.4	[9]	19	0	Affymetrix 500K	[9]		X				
Ireland (IE)	53.2	-8.18	[9]	61	1	Affymetrix 500K	[9]		X				
Italy (IT)	42	12.5	[9]	219	0	Affymetrix 500K	[9]		X				
Kosovo (KS)	42.7	21.1	[9]	2	0	Affymetrix 500K	[9]		X				
Latvia (LV)	56.9	24.9	[9]	1	0	Affymetrix 500K	[9]		X				
Macedonia (MK)	41.7	21.7	[9]	4	0	Affymetrix 500K	[9]		X				
Netherlands (NL)	52.3	5.67	[9]	17	0	Affymetrix 500K	[9]		X				
Norway (NO)	59.9	10.7	[9]	3	0	Affymetrix 500K	[9]		X				
Poland (PL)	52.1	19.4	[9]	22	0	Affymetrix 500K	[9]		X				
Portugal (PT)	39.6	-8.02	[9]	128	0	Affymetrix 500K	[9]		X				
Romania (RO)	45.9	25	[9]	14	0	Affymetrix 500K	[9]		X				
Russia (RU)	55.8	37.5	[9]	6	0	Affymetrix 500K	[9]		X				
Scotland (Sct)	56	-3.2	[9]	5	0	Affymetrix 500K	[9]		X				
Sweden (SE)	59.4	18	[9]	10	0	Affymetrix 500K	[9]		X				
Slovenia (SI)	46.1	14.8	[9]	2	0	Affymetrix 500K	[9]		X				
Slovakia (SK)	48.7	19.5	[9]	1	0	Affymetrix 500K	[9]		X				
Turkey (TR)	39.1	35.4	[9]	4	0	Affymetrix 500K	[9]		X				

Table S2: Populations included in this study (Part II).
