# Supplementary material for: A Quantitative Comparison of the Similarity between Genes and Geography in Worldwide Human Populations
Source: PLoS Genet. 2012 Aug 23;8(8):e1002886. doi: 10.1371/journal.pgen.1002886 (PMC3426559; doi:10.1371/journal.pgen.1002886)
Supplement: Table S3 — Populations included in this study (Part III). (PDF) [file pgen.1002886.s012.pdf]

Population	Latitude (degrees)	Longitude (degrees)	Source of coordinates	Sample size	High-missing-data samples	Genotyping platform	Source of SNP data	Datasets in which the population is included					
								World	Europe	Africa	Asia	E. Asia	C.S. Asia
Ukraine (UA)	49.1	31.4	[9]	1	0	Affymetrix 500K	[9]		X				
Serbia-Montenegro (YG)	43.9	20.6	[9]	44	0	Affymetrix 500K	[9]		X				
Bamoun	5.5	10.8	[37]	20	2	Affymetrix 500K	[26]			X			
Brong	7.5	-2.0	[37]	8	1	Affymetrix 500K	[26]			X			
Bulala	13.0	18.0	[37]	15	0	Affymetrix 500K	[26]			X			
Fang	2.5	13.0	[37]	18	1	Affymetrix 500K	[26]			X			
Hausa	12.0	8.0	[37]	13	2	Affymetrix 500K	[26]			X			
Igbo	6.0	7.0	[37]	17	4	Affymetrix 500K	[26]			X			
Kaba	8.0	16.8	[37]	16	0	Affymetrix 500K	[26]			X			
Kongo	-5.5	15.0	[37]	9	0	Affymetrix 500K	[26]			X			
Mbororo Fulani	11.8	14.8	[37]	13	2	Affymetrix 500K	[26]			X			
Mada	10.8	14.1	[37]	12	0	Affymetrix 500K	[26]			X			
Xhosa	-32.0	28.0	[37]	5	2	Affymetrix 500K	[26]			X			
!Kung	-19.6	20.5	J. Xing	13	0	Affymetrix NspI 250K	[23]			X			
Alur	-3.0	30.9	J. Xing	10	0	Affymetrix NspI 250K	[23]			X			
A.P. Brahmin	17.7	83.3	J. Xing	25	0	Affymetrix NspI 250K	[23]				X		X
A.P. Madiga	17.7	83.3	J. Xing	10	0	Affymetrix NspI 250K	[23]				X		X
A.P. Mala	17.7	83.3	J. Xing	11	0	Affymetrix NspI 250K	[23]				X		X
Bambaran	12.5	-8.0	J. Xing	25	0	Affymetrix 6.0	[23]			X			
Buryat	48.1	114.6	J. Xing	25	0	Affymetrix 6.0	[23]				X	X	
Dogon	15.1	-4.2	J. Xing	24	0	Affymetrix 6.0	[23]			X			
Hema	1.6	30.3	J. Xing	15	0	Affymetrix NspI 250K	[23]			X			
Iban	3.1	113.0	J. Xing	25	0	Affymetrix NspI 250K	[23]				X	X	
Iraqi Kurd	36.7	43.9	J. Xing	24	0	Affymetrix 6.0	[23]				X		
Irula	13.1	80.3	J. Xing	24	0	Affymetrix NspI 250K	[23]				X		X
Kyrgyzstani	43.2	74.6	J. Xing	25	0	Affymetrix 6.0	[23]				X		X
Nepalese	27.7	85.3	J. Xing	25	0	Affymetrix 6.0	[23]				X		X
Nguni	-33.3	26.5	J. Xing	9	0	Affymetrix NspI 250K	[23]			X			
Pakistani	28.6	70.3	J. Xing	25	0	Affymetrix 6.0	[23]				X		X
Pedi	-25.5	26.1	J. Xing	10	0	Affymetrix NspI 250K	[23]			X			
Sotho/Tswana	-26.2	28.1	J. Xing	8	0	Affymetrix NspI 250K	[23]			X			
Stalskoe	43.0	47.5	J. Xing	5	0	Affymetrix NspI 250K	[23]				X		
Thai	7.9	98.3	J. Xing	24	0	Affymetrix 6.0	[23]				X	X	
T.N. Brahmin	13.1	80.3	J. Xing	14	0	Affymetrix NspI 250K	[23]				X		X
T.N. Dalit	13.1	80.3	J. Xing	13	0	Affymetrix 6.0	[23]				X		X
Urkarah	43.0	47.5	J. Xing	18	0	Affymetrix NspI 250K	[23]				X		
Vietnamese	16.7	107.3	J. Xing	7	0	Affymetrix NspI 250K	[23]				X	X	
Tibetan	34.9	98.2	[32]	31	0	Affymetrix 6.0	[32]				X	X	

Table S3: Populations included in this study (Part III).
