# Supplementary material for: A Quantitative Comparison of the Similarity between Genes and Geography in Worldwide Human Populations
Source: PLoS Genet. 2012 Aug 23;8(8):e1002886. doi: 10.1371/journal.pgen.1002886 (PMC3426559; doi:10.1371/journal.pgen.1002886)
Supplement: Table S4 — Change of the Procrustes similarity when excluding one population from the worldwide example. (PDF) [file pgen.1002886.s013.pdf]

| Population excluded | Number of individuals excluded | Similarity to original PCA $t'$ | Similarity to geography $t''$ | $t'' - t_0$ |
|---|---|---|---|---|
| Han | 34 | 1.000 | 0.715 | 0.010 |
| Maya | 21 | 1.000 | 0.713 | 0.008 |
| Karitiana | 13 | 1.000 | 0.710 | 0.005 |
| Xibo | 9 | 1.000 | 0.710 | 0.005 |
| Dai | 10 | 1.000 | 0.708 | 0.003 |
| Yi | 10 | 1.000 | 0.708 | 0.003 |
| Tujia | 10 | 1.000 | 0.708 | 0.003 |
| Miao | 10 | 1.000 | 0.708 | 0.003 |
| Tu | 10 | 1.000 | 0.707 | 0.002 |
| Naxi | 8 | 1.000 | 0.707 | 0.002 |
| Lahu | 8 | 1.000 | 0.707 | 0.002 |
| Surui | 8 | 1.000 | 0.707 | 0.002 |
| Sindhi | 24 | 1.000 | 0.707 | 0.002 |
| Makrani | 25 | 1.000 | 0.707 | 0.002 |
| Mongola | 10 | 1.000 | 0.707 | 0.002 |
| Yakut | 25 | 1.000 | 0.707 | 0.002 |
| Han (N. China) | 10 | 1.000 | 0.707 | 0.002 |
| She | 10 | 1.000 | 0.707 | 0.002 |
| Hazara | 22 | 1.000 | 0.707 | 0.002 |
| Brahui | 25 | 1.000 | 0.707 | 0.002 |
| Cambodian | 10 | 1.000 | 0.707 | 0.002 |
| Papuan | 17 | 1.000 | 0.707 | 0.002 |
| Japanese | 28 | 1.000 | 0.707 | 0.002 |
| Balochi | 24 | 1.000 | 0.707 | 0.002 |
| Daur | 9 | 1.000 | 0.706 | 0.001 |
| Colombian | 7 | 1.000 | 0.706 | 0.001 |
| Oroqen | 9 | 1.000 | 0.706 | 0.001 |
| Melanesian | 11 | 1.000 | 0.706 | 0.001 |
| Pathan | 22 | 1.000 | 0.706 | 0.001 |
| Kalash | 23 | 1.000 | 0.706 | 0.001 |
| Hezhen | 9 | 1.000 | 0.706 | 0.001 |
| Mandenka | 22 | 1.000 | 0.705 | 0.000 |
| Uygur | 10 | 1.000 | 0.705 | 0.000 |
| Burusho | 25 | 1.000 | 0.705 | 0.000 |
| Yoruba | 21 | 1.000 | 0.704 | -0.001 |
| Tuscan | 7 | 1.000 | 0.704 | -0.001 |
| Druze | 42 | 1.000 | 0.704 | -0.001 |
| Adygei | 17 | 1.000 | 0.704 | -0.001 |
| Biaka Pygmy | 22 | 1.000 | 0.703 | -0.002 |
| Italian | 12 | 1.000 | 0.703 | -0.002 |
| Mbuti Pygmy | 13 | 1.000 | 0.703 | -0.002 |
| Orcadian | 15 | 1.000 | 0.703 | -0.002 |
| Basque | 24 | 1.000 | 0.702 | -0.003 |
| Russian | 25 | 1.000 | 0.702 | -0.003 |
| French | 28 | 1.000 | 0.702 | -0.003 |
| Palestinian | 46 | 1.000 | 0.701 | -0.004 |
| Bantu (Kenya) | 11 | 1.000 | 0.701 | -0.004 |
| Bedouin | 45 | 1.000 | 0.701 | -0.004 |
| Sardinian | 28 | 1.000 | 0.701 | -0.004 |
| San | 5 | 1.000 | 0.700 | -0.005 |
| Pima | 14 | 1.000 | 0.700 | -0.005 |
| Mozabite | 27 | 1.000 | 0.699 | -0.006 |
| Bantu (S. Africa) | 8 | 1.000 | 0.697 | -0.008 |

Table S4: Change of the Procrustes similarity when excluding one population from the worldwide example. The Procrustes similarity between genetic coordinates and geographic coordinates is $t_0 = 0.705$ in the original analysis (Fig. 1).
