# Supplementary material for: A Quantitative Comparison of the Similarity between Genes and Geography in Worldwide Human Populations
Source: PLoS Genet. 2012 Aug 23;8(8):e1002886. doi: 10.1371/journal.pgen.1002886 (PMC3426559; doi:10.1371/journal.pgen.1002886)
Supplement: Table S5 — Change of the Procrustes similarity when excluding one population from the European example. (PDF) [file pgen.1002886.s014.pdf]

Population excluded	Number of individuals excluded	Similarity to original PCA t'	Similarity to geography t''	$t'' - t_0$
Italy (IT)	219	0.986	0.810	0.030
Russia (RU)	6	1.000	0.788	0.008
Swiss-French (CH-F)	125	1.000	0.785	0.005
Swiss-German (CH-G)	84	1.000	0.785	0.005
Germany (DE)	69	1.000	0.783	0.003
France (FR)	89	1.000	0.783	0.003
Sweden (SE)	10	1.000	0.782	0.002
Swiss-Italian (CH-I)	13	1.000	0.781	0.001
Austria (AT)	14	1.000	0.781	0.001
Slovakia (SK)	1	1.000	0.780	0.000
Hungary (HU)	19	1.000	0.780	0.000
Romania (RO)	14	1.000	0.780	0.000
Finland (FI)	1	1.000	0.780	0.000
Ukraine (UA)	1	1.000	0.780	0.000
Bulgaria (BG)	2	1.000	0.780	0.000
Slovenia (SI)	2	1.000	0.779	-0.001
Denmark (DK)	1	1.000	0.779	-0.001
Latvia (LV)	1	1.000	0.779	-0.001
Norway (NO)	3	1.000	0.779	-0.001
Poland (PL)	22	0.999	0.779	-0.001
Turkey (TR)	4	1.000	0.779	-0.001
Croatia (HR)	8	1.000	0.779	-0.001
Kosovo (KS)	2	1.000	0.779	-0.001
Belgium (BE)	42	1.000	0.779	-0.001
Czech Republic (CZ)	11	1.000	0.779	-0.001
Cyprus (CY)	4	1.000	0.779	-0.001
Scotland (Sct)	5	1.000	0.779	-0.001
Netherlands (NL)	17	1.000	0.779	-0.001
Macedonia (MK)	4	1.000	0.779	-0.001
Albania (AL)	3	1.000	0.779	-0.001
Bosnia-Herzegovina (BA)	9	1.000	0.779	-0.001
Greece (GR)	8	1.000	0.778	-0.002
Ireland (IE)	60	0.999	0.776	-0.004
Serbia-Montenegro (YG)	44	0.998	0.772	-0.008
Spain (ES)	136	0.994	0.770	-0.010
Portugal (PT)	126	0.990	0.769	-0.011
United Kingdom (GB)	199	0.998	0.764	-0.016

Table S5: Change of the Procrustes similarity when excluding one population from the European example. The Procrustes similarity between genetic coordinates and geographic coordinates is $t_0 = 0.780$ in the original analysis (Fig. 2).
