# Supplementary material for: A Quantitative Comparison of the Similarity between Genes and Geography in Worldwide Human Populations
Source: PLoS Genet. 2012 Aug 23;8(8):e1002886. doi: 10.1371/journal.pgen.1002886 (PMC3426559; doi:10.1371/journal.pgen.1002886)
Supplement: Table S6 — Change of the Procrustes similarity when excluding one population from the Sub-Saharan African example. (PDF) [file pgen.1002886.s015.pdf]

Population excluded	Number of individuals excluded	Similarity to original PCA t'	Similarity to geography t''	$t'' - t_0$
Maasai (MKK)	30	0.980	0.832	0.042
Luhya (LWK)	30	0.999	0.808	0.018
Bamoun	18	1.000	0.797	0.007
Bantu (Kenya)	11	1.000	0.797	0.007
Fang	17	1.000	0.796	0.006
Mandenka	22	0.999	0.795	0.005
Kaba	16	1.000	0.794	0.004
Hausa	13	1.000	0.794	0.004
Igbo	17	1.000	0.791	0.001
Kongo	9	1.000	0.791	0.001
Yoruba	21	1.000	0.791	0.001
Alur	10	1.000	0.789	-0.001
Brong	7	1.000	0.788	-0.002
Dogon	24	0.995	0.788	-0.002
Bambaran	25	0.999	0.786	-0.004
Mada	12	1.000	0.785	-0.005
Hema	13	1.000	0.784	-0.006
Xhosa	3	1.000	0.783	-0.007
Bantu (S. Africa)	8	0.999	0.781	-0.009
Bulala	15	0.999	0.780	-0.010
Pedi	10	0.998	0.775	-0.015
Nguni	9	0.998	0.774	-0.016
Sotho/Tswana	8	0.997	0.768	-0.022

Table S6: Change of the Procrustes similarity when excluding one population from the Sub-Saharan African example. The Procrustes similarity between genetic coordinates and geographic coordinates is $t_0 = 0.790$ in the original analysis (Fig. 3).
