# Supplementary material for: A Quantitative Comparison of the Similarity between Genes and Geography in Worldwide Human Populations
Source: PLoS Genet. 2012 Aug 23;8(8):e1002886. doi: 10.1371/journal.pgen.1002886 (PMC3426559; doi:10.1371/journal.pgen.1002886)
Supplement: Table S7 — Summary of the results for Sub-Saharan Africa when all or one of five additional African populations are included (corresponding to Figure S3). (PDF) [file pgen.1002886.s016.pdf]

Populations added	Panel in Fig. S3	Number of populations	Number of individuals collected	Number of individuals in the analysis	Variance explained by PC1 (%)	Variance explained by PC2 (%)	Rotation angle θ (°)	Procrustes similarity t_0	P-value of t_0	F_{ST} (%)
All	B	28	422	412	1.68	1.21	-78.47	0.548	0.00040	2.567
Mbororo Fulani	C	24	369	361	1.40	0.84	29.25	0.605	0.00005	1.518
Biaka Pygmy	D	24	378	369	1.26	1.03	20.01	0.559	0.00278	1.652
Mbuti Pygmy	E	24	369	359	1.27	1.19	-10.05	0.543	0.00120	1.781
!Kung	F	24	369	361	1.29	1.04	3.89	0.721	$< 10^{-5}$	1.616
San	G	24	361	354	1.31	0.92	5.66	0.725	$< 10^{-5}$	1.578

Table S7: Summary of the results for Sub-Saharan Africa when all or one of five additional African populations are included (corresponding to Fig. S3). θ is the rotation angle for the PCA map that optimizes the Procrustes similarity with the geographic map, and it is measured in degrees counterclockwise. P-values are obtained from 100,000 permutations of population labels.
