# Supplementary material for: A Quantitative Comparison of the Similarity between Genes and Geography in Worldwide Human Populations
Source: PLoS Genet. 2012 Aug 23;8(8):e1002886. doi: 10.1371/journal.pgen.1002886 (PMC3426559; doi:10.1371/journal.pgen.1002886)
Supplement: Table S8 — Change of the Procrustes similarity when excluding one population from the Asian example. (PDF) [file pgen.1002886.s017.pdf]

Population excluded	Number of individuals excluded	Similarity to original PCA t'	Similarity to geography t''	$t'' - t_0$
Irula	24	0.993	0.871	0.022
Xibo	9	1.000	0.857	0.008
Tibetan	31	1.000	0.854	0.005
Kyrgyzstani	25	1.000	0.854	0.005
A.P. Brahmin	25	1.000	0.854	0.005
Nepalese	25	1.000	0.853	0.004
Yakut	25	0.999	0.853	0.004
T.N. Dalit	13	1.000	0.853	0.004
A.P. Mala	11	1.000	0.852	0.003
Hazara	22	1.000	0.852	0.003
A.P. Madiga	10	1.000	0.852	0.003
Naxi	8	1.000	0.852	0.003
T.N. Brahmin	14	1.000	0.851	0.002
Lahu	8	1.000	0.851	0.002
Yi	10	1.000	0.851	0.002
Dai	10	1.000	0.850	0.001
Tu	10	1.000	0.850	0.001
Thai	24	1.000	0.850	0.001
Uygur	10	1.000	0.849	0.000
Vietnamese	7	1.000	0.849	0.000
Tujia	10	1.000	0.849	0.000
Miao	10	1.000	0.849	0.000
Kalash	23	1.000	0.849	0.000
Stalskoe	5	1.000	0.849	0.000
Burusho	25	1.000	0.848	-0.001
Han (N. China)	10	1.000	0.848	-0.001
Iban	25	0.999	0.848	-0.001
Cambodian	10	1.000	0.848	-0.001
Pathan	22	1.000	0.847	-0.002
Hezhen	9	1.000	0.847	-0.002
She	10	1.000	0.847	-0.002
Mongola	10	1.000	0.847	-0.002
Makrani	20	1.000	0.847	-0.002
Balochi	22	1.000	0.847	-0.002
Japanese	28	1.000	0.847	-0.002
Brahui	23	1.000	0.847	-0.002
Daur	9	1.000	0.846	-0.003
Pakistani	25	1.000	0.846	-0.003
Sindhi	22	1.000	0.846	-0.003
Oroqen	9	1.000	0.846	-0.003
Urkarah	18	1.000	0.845	-0.004
Iraqi Kurd	24	1.000	0.845	-0.004
Han	34	1.000	0.844	-0.005
Buryat	25	1.000	0.839	-0.010

Table S8: Change of the Procrustes similarity when excluding one population from the Asian example. The Procrustes similarity between genetic coordinates and geographic coordinates is $t_0 = 0.849$ in the original analysis (Fig. 4).
