# Supplementary material for: A Quantitative Comparison of the Similarity between Genes and Geography in Worldwide Human Populations
Source: PLoS Genet. 2012 Aug 23;8(8):e1002886. doi: 10.1371/journal.pgen.1002886 (PMC3426559; doi:10.1371/journal.pgen.1002886)
Supplement: Table S9 — Change of the Procrustes similarity when excluding one population from the East Asian example. (PDF) [file pgen.1002886.s018.pdf]

Population excluded	Number of individuals excluded	Similarity to original PCA t'	Similarity to geography t''	$t'' - t_0$
Japanese	28	0.999	0.755	0.115
Thai	20	0.994	0.691	0.051
Han	34	0.999	0.673	0.033
Xibo	8	1.000	0.655	0.015
Tibetan	31	0.996	0.655	0.015
She	10	1.000	0.654	0.014
Hezhen	9	1.000	0.645	0.005
Han (N. China)	10	1.000	0.645	0.005
Miao	10	1.000	0.642	0.002
Tujia	10	1.000	0.642	0.002
Mongola	10	1.000	0.640	0.000
Dai	10	1.000	0.637	-0.003
Vietnamese	7	1.000	0.637	-0.003
Tu	10	1.000	0.637	-0.003
Lahu	8	1.000	0.636	-0.004
Daur	9	1.000	0.636	-0.004
Cambodian	10	1.000	0.635	-0.005
Buryat	25	0.999	0.635	-0.005
Naxi	8	1.000	0.634	-0.006
Yi	10	1.000	0.631	-0.009
Oroqen	9	1.000	0.631	-0.009
Yakut	23	0.988	0.577	-0.063
Iban	25	0.993	0.561	-0.079

Table S9: Change of the Procrustes similarity when excluding one population from the East Asian example. The Procrustes similarity between genetic coordinates and geographic coordinates is $t_0 = 0.640$ in the original analysis (Fig. 5).
