# Supplementary material for: A Quantitative Comparison of the Similarity between Genes and Geography in Worldwide Human Populations
Source: PLoS Genet. 2012 Aug 23;8(8):e1002886. doi: 10.1371/journal.pgen.1002886 (PMC3426559; doi:10.1371/journal.pgen.1002886)
Supplement: Table S10 — Change of the Procrustes similarity when excluding one population from the Central/South Asian example. (PDF) [file pgen.1002886.s019.pdf]

Population excluded	Number of individuals excluded	Similarity to original PCA t'	Similarity to geography t''	$t'' - t_0$
Hazara	22	1.000	0.769	0.032
Kalash	23	1.000	0.754	0.017
A.P. Brahmin	25	1.000	0.749	0.012
T.N. Brahmin	14	1.000	0.748	0.011
Nepalese	25	1.000	0.747	0.010
Burusho	25	1.000	0.747	0.010
Pathan	22	1.000	0.740	0.003
Pakistani	25	1.000	0.736	-0.001
Sindhi	22	1.000	0.732	-0.005
A.P. Madiga	10	1.000	0.724	-0.013
Uygur	10	1.000	0.723	-0.014
A.P. Mala	11	1.000	0.721	-0.016
Kyrgyzstani	25	0.992	0.720	-0.017
Balochi	23	0.999	0.720	-0.017
T.N. Dalit	13	0.999	0.720	-0.017
Brahui	23	0.999	0.718	-0.019
Makrani	20	0.999	0.718	-0.019
Irula	24	0.979	0.717	-0.020

Table S10: Change of the Procrustes similarity when excluding one population from the Central/South Asian example. The Procrustes similarity between genetic coordinates and geographic coordinates is $t_0 = 0.737$ in the original analysis (Fig. 6).
