# Supplementary material for: A Quantitative Comparison of the Similarity between Genes and Geography in Worldwide Human Populations
Source: PLoS Genet. 2012 Aug 23;8(8):e1002886. doi: 10.1371/journal.pgen.1002886 (PMC3426559; doi:10.1371/journal.pgen.1002886)
Supplement: Table S11 — Samples identified as PCA outliers in the analyses for different geographic regions. (PDF) [file pgen.1002886.s020.pdf]

Analysis	Region	Number of PCA outliers	Sample ID of PCA outliers	Population
Fig. 1	Worldwide	0	-	-
Fig. 2	Europe	2	POPR26466<br>POPR48136	Portugal (PT)<br>Portugal (PT)
Fig. 3	Sub-Saharan Africa	2	AFH7<br>AFH10	Hema<br>Hema
Fig. 4	Asia	11	HGDP00057<br>HGDP00060<br>HGDP00013<br>HGDP00029<br>HGDP00130<br>HGDP00139<br>HGDP00150<br>HGDP00154<br>HGDP00157<br>HGDP00173<br>HGDP00175	Balochi<br>Balochi<br>Brahui<br>Brahui<br>Makrani<br>Makrani<br>Makrani<br>Makrani<br>Makrani<br>Sindhi<br>Sindhi
Fig. 5	East Asia	7	F066579<br>F066599<br>F066607<br>F066612<br>HGDP01243<br>HGDP00949<br>HGDP00953	Thai<br>Thai<br>Thai<br>Thai<br>Xibo<br>Yakut<br>Yakut
Fig. 6	Central/South Asia	10	HGDP00057<br>HGDP00013<br>HGDP00029<br>HGDP00130<br>HGDP00150<br>HGDP00151<br>HGDP00154<br>HGDP00157<br>HGDP00173<br>HGDP00175	Balochi<br>Brahui<br>Brahui<br>Makrani<br>Makrani<br>Makrani<br>Makrani<br>Makrani<br>Sindhi<br>Sindhi
Fig. S3B	Sub-Saharan Africa	4	AFH7<br>AFH10<br>NA21417<br>NA21596	Hema<br>Hema<br>Maasai (MKK)<br>Maasai (MKK)
Fig. S3C	Sub-Saharan Africa	2	AFH7<br>AFH10	Hema<br>Hema
Fig. S3D	Sub-Saharan Africa	3	AFH7<br>AFH10<br>NA21417	Hema<br>Hema<br>Maasai (MKK)
Fig. S3E	Sub-Saharan Africa	4	AFH7<br>AFH10<br>NA21417<br>NA21596	Hema<br>Hema<br>Maasai (MKK)<br>Maasai (MKK)
Fig. S3F	Sub-Saharan Africa	2	NA21417<br>TSW25	Maasai (MKK)<br>Sotho/Tswana
Fig. S3G	Sub-Saharan Africa	1	NA21417	Maasai (MKK)

Table S11: Samples identified as PCA outliers in the analyses for different geographic regions. Note that AFH7 and AFH10, which appeared as PCA outliers in most of the Sub-Saharan African examples, are likely to be relatives based on allele-sharing analysis (results not shown).
